# Exploratory Associations Between Multimodal MRI-Derived Features and Neurological Symptoms in Wolfram Syndrome: A Spanish Cohort Pilot Study

**DOI:** 10.3390/diagnostics16152396

**Published:** 2026-07-30

**Authors:** Gema Esteban-Bueno, Lucas Fernández-Brillet, Juan Luis Fernández-Martínez

**Affiliations:** 1 UGC La Cañada (Primary Care Management Unit), Almería Periphery–Almería Health District, Andalusian Health Service (SAS), 04009 Almería, Spain; 2 Spanish Association for Research and Support to Wolfram Syndrome, 04120 Almería, Spain; 3 Miscellany Creative SL, 29620 Málaga, Spain; miscellany.creative.sl@gmail.com; 4 Group of Inverse Problems, Optimization and Machine Learning, Department of Mathematics, University of Oviedo, 33007 Oviedo, Spain; jlfm@uniovi.es

**Keywords:** wolfram syndrome, WFS1, multimodal MRI, diffusion tensor imaging, FLAIR, neurodegeneration, imaging correlates, rare diseases, neurological involvement, quantitative MRI

## Abstract

**Background/Objectives**: Wolfram syndrome is an ultra-rare, progressive multisystem disorder in which endocrine and sensory manifestations coexist with neurological involvement. Quantitative magnetic resonance imaging (MRI) may help characterize central nervous system involvement in this condition; however, evidence derived from small imaging cohorts requires cautious interpretation. This study aimed to examine the relationships between different MRI-derived attributes and neurological symptoms in Wolfram syndrome, with the goal of identifying exploratory imaging patterns that may suggest the involvement of specific neural systems. **Methods:** We analyzed a Spanish cohort of 45 genetically confirmed patients with Wolfram syndrome. A homogeneous subset of 15 patients with standardized 3-Tesla multimodal MRI and adequate image quality was included in the quantitative imaging analysis. T1-weighted MRI, T2-weighted/fluid-attenuated inversion recovery (FLAIR) imaging, and diffusion tensor imaging (DTI) were processed using a standardized workflow for brain extraction, anatomical segmentation, cortical reconstruction, and quantitative feature extraction. A total of 172 MRI-derived features were examined in relation to neurological phenotypes, including dysphagia, ataxia, gait instability, and cognitive impairment. Analyses included principal component analysis, exploratory factor analysis, correlation analyses, and symptom-specific group comparisons. Given the small MRI sample size and the high feature-to-subject ratio, all analyses were considered exploratory and hypothesis-generating, and the findings should be interpreted cautiously pending validation in larger, independent cohorts. **Results:** Multimodal MRI-derived features showed distributed associations with neurological manifestations. The most recurrent exploratory imaging correlates involved the thalamus, lateral geniculate nucleus, cerebellum, brainstem, ventricular system, corpus callosum, posterior cortical regions, and white-matter pathways. FLAIR-derived signal heterogeneity in the thalamus and lateral geniculate nucleus appeared repeatedly across several clinical manifestations. Dysphagia was associated with a distributed pattern involving cortical thinning, thalamic and brainstem volume reduction, reduced cerebellar white-matter integrity, increased FLAIR heterogeneity, and ventricular enlargement. Ataxia and gait instability showed overlapping but partially distinct imaging profiles, whereas cognitive impairment was associated with broader cortical, subcortical, callosal, cerebellar, and ventricular alterations. **Conclusions:** In this exploratory pilot study, multimodal MRI-derived features showed clinically plausible associations with neurological manifestations in Wolfram syndrome. The findings support a distributed model of neurological involvement affecting cerebello-thalamo-cortical circuits, visual relay structures, brainstem pathways, and long-range white-matter connections. These results should be interpreted as exploratory MRI-derived attributes rather than as validated biomarkers, prognostic indicators, or clinically applicable imaging signatures. Confirmation in future longitudinal, multicenter studies with harmonized imaging protocols and external validation will be required.

## 1. Introduction

Wolfram syndrome (WS) is an ultra-rare, progressive neurodegenerative and endocrine disorder classically defined by diabetes mellitus, optic atrophy, diabetes insipidus, and sensorineural hearing loss. Its estimated prevalence has been reported to range approximately from 1 in 500,000 to 1 in 770,000 individuals, although epidemiological estimates vary because of underdiagnosis, founder effects, and the limited number of reported cohorts. No consistent sex or racial predilection has been established, and cases have been described across different ethnic and geographic backgrounds. The clinical spectrum extends beyond the historical DIDMOAD acronym and includes urological dysfunction, psychiatric manifestations, bulbar symptoms, ataxia, gait impairment, and cognitive involvement [1,2,3,4,5,6,7]. These neurological features are major determinants of disability, quality of life, and prognosis, particularly when brainstem and cerebellar systems become involved.

Most cases are caused by biallelic pathogenic variants in WFS1, which encodes the endoplasmic reticulum protein wolframin. Less frequently, a WS-like phenotype is associated with pathogenic variants in CISD2, which encodes CDGSH iron–sulfur domain-containing protein 2, a protein involved in endoplasmic reticulum–mitochondrial homeostasis and cellular stress regulation. Pathogenic CISD2 variants are associated with Wolfram syndrome type 2 or WS-like phenotypes, which may overlap clinically with WFS1-related disease but can show distinct systemic features. The genetic heterogeneity of WS and WFS1-related disorders contributes to marked variability in age at onset, symptom sequence, neurological severity, and rate of progression [8,9,10,11,12]. This heterogeneity reinforces the need for objective quantitative imaging measures that can complement clinical examination and help characterize central nervous system (CNS) involvement.

From a clinical perspective, neurological assessment remains central to patient follow-up, but it has important limitations in an ultra-rare progressive disease. Symptoms such as dysphagia, dysarthria, ataxia, gait instability, abnormal tandem gait, anosmia, and cognitive impairment may emerge gradually, fluctuate across visits, or become clinically evident only after substantial tissue damage has occurred. Quantitative MRI-derived imaging measures may therefore provide clinically useful information by supporting the characterization of subclinical or early CNS involvement, estimating neurological burden, and supporting individualized follow-up intervals [3,5,13].

Magnetic resonance imaging (MRI) provides a non-invasive framework for evaluating structural, signal-based, and microstructural brain alterations in WS. Previous neuroimaging studies have shown that brain involvement is not restricted to the cerebellum or brainstem, but affects a broader network that includes the thalamus, optic pathways, white matter, ventral pons, cerebellum, and posterior cortical regions [14,15,16,17,18,19,20]. Diffusion MRI studies have further suggested abnormal myelination and impaired white-matter integrity, while longitudinal morphometric analyses support a combined pattern of altered neurodevelopment and progressive neurodegeneration [15,17,18,21].

In routine clinical practice, MRI interpretation in WS has traditionally emphasized visible features such as optic nerve atrophy, brainstem and cerebellar atrophy, posterior pituitary abnormalities, and white-matter signal changes. However, quantitative multimodal MRI may capture more subtle changes, including regional cortical thinning, ventricular enlargement, FLAIR signal heterogeneity, diffusion abnormalities, and interregional ratios that are difficult to evaluate visually. Such features are particularly relevant for diseases in which small cohorts limit conventional statistical modeling. The use of reproducible quantitative imaging features and standardized quantitative imaging feature definitions is also important for improving comparability across small-cohort MRI studies [22,23].

Machine learning and multivariate analytical approaches are increasingly used in neuroimaging to integrate high-dimensional structural, diffusion, and signal-intensity features. These methods can support the identification of exploratory imaging patterns in neurological diseases. However, in ultra-rare conditions, their application should prioritize interpretability, dimensionality reduction, biological plausibility, and cautious validation, rather than apparent predictive performance in small datasets [24,25].

This study aimed to examine the relationships between different MRI-derived attributes and neurological symptoms in Wolfram syndrome, with the goal of identifying exploratory imaging patterns that may suggest the involvement of specific neural systems. We assessed whether volumetric, cortical, FLAIR-derived, diffusion-based, normalized, and asymmetry features showed clinically plausible associations with neurological manifestations, including dysphagia, ataxia, gait instability, and cognitive impairment. Given the ultra-rare nature of the disease, the small quantitative MRI subset, and the high feature-to-subject ratio, this study was designed as an exploratory, hypothesis-generating analysis rather than as a biomarker validation study. This framework applies to all analyses reported below; therefore, the exploratory nature of the findings is not restated in every subsection. All MRI-derived associations should be interpreted within this context and require validation in larger, independent cohorts with harmonized MRI acquisition protocols before biomarker-level or clinical interpretation can be supported.

## 2. Materials and Methods

### 2.1. Study Design, Setting, and Participants

This observational cohort-based study included a Spanish series of 45 patients with genetically confirmed Wolfram syndrome and available clinical follow-up information. Clinical data were used to characterize neurological involvement and define outcome variables for association analyses. For quantitative neuroimaging, a homogeneous subset of 15 patients was selected according to the availability of a standardized multimodal MRI protocol and sufficient image quality for automated processing. Patients were not selected based on neurological severity, but according to MRI protocol availability and image quality. MRI examinations in this quantitative subset were acquired on a clinical 3-Tesla Ingenia DStream MRI system (Philips Medical Systems Nederland B.V., Best, The Netherlands) at Clínica Tecnológica Médica, Almería, Spain, and included T1-weighted structural MRI, T2/FLAIR imaging, and diffusion tensor imaging (DTI). The examinations were obtained as part of clinical care and were subsequently used for retrospective quantitative analysis. Complete acquisition metadata, including coil configuration and full sequence parameters, were not available for all examinations, and this is acknowledged as a methodological limitation. Given the limited sample size, the study was designed as an exploratory, hypothesis-generating analysis of clinical–imaging associations, rather than as a diagnostic, prognostic, or biomarker-validation study. Within this framework, the methodology was intended to identify clinically and anatomically plausible relationships between MRI-derived attributes and different neurological manifestations in patients with Wolfram syndrome.

### 2.2. Clinical Phenotyping and Neurological Outcomes

The clinical analysis focused on neurological manifestations with direct relevance to patient function and disease monitoring. Available outcomes included dysphagia, sialorrhea, absent gag reflex, dysarthria, dysmetria, adiadochokinesia, ataxia, gait instability, impaired tandem gait, anosmia, hyperreflexia, and cognitive impairment. Neurological phenotypes were extracted from the available longitudinal clinical follow-up records and were based on clinical examination, documented neurological signs, and patient- or caregiver-reported symptoms when clinically relevant. For symptom-specific analyses, phenotypes were coded according to their presence or absence in the available clinical dataset. Binary coding was used because uniform severity scales, instrumental swallowing assessments, and standardized neuropsychological instruments were not available for all patients across the full cohort. This approach was selected to maximize interpretability in an ultra-rare disease cohort while avoiding overfitting from excessive outcome granularity. However, binary coding does not capture symptom severity, progression, or temporal variability and may therefore limit the precision of the phenotype definitions.

Dysphagia was considered a clinically high-risk manifestation because it may reflect bulbar and corticobulbar involvement and can be associated with aspiration, nutritional compromise, and respiratory complications. Dysphagia status was coded from clinical records when swallowing difficulty, choking episodes, aspiration risk, dietary modification, or clinically documented swallowing impairment was reported. Ataxia and gait instability were interpreted as motor-coordination phenotypes potentially involving cerebellar, thalamic, proprioceptive, and long-range white-matter pathways. Ataxia, dysmetria, adiadochokinesia, impaired tandem gait, and gait instability were coded according to documented neurological examination findings and follow-up notes. Cognitive impairment was considered a marker of broader cortical–subcortical network involvement rather than an isolated domain-specific deficit.

Cognitive impairment was coded when cognitive difficulties or impairment were documented in the clinical follow-up records; because a uniform neuropsychological battery was not available for all patients, this outcome was interpreted as an exploratory clinical phenotype rather than as a formally quantified cognitive endpoint.

Clinical phenotyping was performed independently of the quantitative MRI feature extraction. MRI-derived analyses were subsequently related to these predefined clinical variables. Given the retrospective nature of the clinical dataset and the small imaging subset, all clinical–imaging associations were interpreted as exploratory and hypothesis-generating.

### 2.3. MRI Preprocessing

MRI data were processed using a standardized multimodal workflow designed to improve consistency across subjects and support reproducible feature extraction. MRI examinations were obtained as part of clinical care and were subsequently used for retrospective quantitative analysis. Quantitative MRI preprocessing, feature extraction, and quality control were performed by the image-analysis team.

For the MRI examinations acquired at Clínica Tecnológica Médica Almería, routine onsite acquisition checks included verification of sequence completeness, anatomical coverage, motion artifacts, and other visible acquisition-related problems.

Image segmentation and visual inspection of the preprocessing outputs were performed by L.F.-B., PhD in Artificial Intelligence, as part of the image-analysis workflow. This technical quality-control procedure included assessment of anatomical alignment, segmentation boundaries, preprocessing failures, and the suitability of the preprocessing outputs for quantitative parameter extraction.

The extracted imaging parameters and resulting quantitative analyses were subsequently reviewed in collaboration with J.L.F.-M., Full Professor of Applied Mathematics, and G.E.-B., MD, who provided medical oversight and assessed the anatomical and clinical plausibility of the results. Thus, quality control was based on a multidisciplinary review, while no independent blinded neuroradiological adjudication was performed. Therefore, the imaging results are presented as exploratory quantitative imaging attributes rather than as conventional neuroradiological diagnoses or validated imaging biomarkers. External validation in larger cohorts will be required for that purpose.

Structural T1-weighted images were processed with FreeSurfer 7.4.1, which includes intensity normalization, skull stripping, automated anatomical segmentation, cortical surface reconstruction, and extraction of cortical and subcortical morphometric measures. The main outputs used for quality control and feature derivation included the normalized T1 image, the brain mask, the automated anatomical segmentation, and the reconstructed white and pial cortical surfaces.

Preprocessing also included spatial-orientation standardization, intensity harmonization, brain extraction, anatomical segmentation, and cortical surface reconstruction. Segmentation outputs were used to identify cortical, subcortical, cerebellar, brainstem, ventricular, and white-matter compartments. Quality control focused on the adequacy of image quality, brain extraction, anatomical segmentation, and cortical surface reconstruction. Quality control was based on visual inspection of preprocessing outputs, including the brain mask, gross anatomical segmentation, cortical surface reconstruction, and the exclusion of scans with major motion artifacts or clear automated-processing failure. Scans with insufficient quality for automated processing were not included in the homogeneous quantitative imaging subset. Because the study was retrospective, complete acquisition metadata and full harmonized quality-control metrics were not available for all examinations, which is acknowledged as a methodological limitation.

### 2.4. MRI-Derived Feature Extraction

A multimodal set of quantitative MRI-derived features was extracted from predefined anatomical regions and sequence-specific outputs, following the general principles of quantitative imaging feature extraction [22,23]. The extracted variables were interpreted as exploratory MRI-derived imaging features rather than as validated imaging biomarkers. Structural features included regional volumes of the thalamus, lateral geniculate nucleus, brainstem, pons, cerebellum, cortical gray matter, corpus callosum, and ventricular system. Because the lateral geniculate nucleus is a small structure and its segmentation on conventional MRI can be technically challenging, LGN-derived measures were interpreted with caution. Cortical measures included regional gray-matter volume, cortical thickness, and surface-based morphometric descriptors in motor, parietal, occipital, and insular cortices.

FLAIR-derived features were extracted to characterize signal abnormalities and tissue heterogeneity. These included mean intensity, standard deviation, coefficient of variation, percentile-based measures, and regional z-score-derived signal descriptors, which were interpreted as exploratory signal-heterogeneity markers [22,23]. DTI-derived features included fractional anisotropy and mean diffusivity, which were used as exploratory imaging measures of white-matter and regional microstructural integrity at global, cerebellar, brainstem, thalamic, and visual-relay levels. No MRI-derived feature was considered independently diagnostic or prognostic in the absence of external validation.

### 2.5. Feature Normalization and Data Integration

To reduce inter-subject variability unrelated to neurological involvement, regional volumetric measures were normalized by intracranial volume when appropriate. Diffusion- and FLAIR-derived measures were additionally normalized against global white-matter reference values when this improved comparability across subjects. Derived features included hemispheric asymmetry indices, intracranial volume-normalized measures, and ratios between anatomically related structures, including lateral geniculate nucleus/thalamus ratios. The final imaging dataset consisted of a 15 × 172 feature matrix integrating structural, FLAIR-based, diffusion-derived, normalized, and ratio-based measures. Given the high feature-to-subject ratio, this integrated dataset was used for exploratory pattern discovery and hypothesis generation rather than for confirmatory predictive modeling or biomarker validation.

### 2.6. Statistical and Exploratory Modeling Strategy

Given the small imaging cohort and the high dimensionality of the feature space, the analysis was designed as exploratory and hypothesis-generating. The primary objective was not to build a definitive diagnostic classifier, prognostic model, or validated biomarker panel, but to identify interpretable MRI-derived patterns that could be tested as exploratory imaging correlates in future studies. Dimensionality reduction was applied using principal component analysis (PCA) and factor analysis to summarize correlated imaging measures and reduce redundancy. All statistical and exploratory analyses were performed using custom scripts developed in Python version 3.14.6 (Python Software Foundation). Principal component analysis (PCA) was performed using scikit-learn version 1.9.0, whereas exploratory factor analysis (EFA) with oblimin rotation was conducted using factor_analyzer version 0.5.1. Correlation analyses, Fisher-ratio calculations, bootstrap resampling, and sensitivity analyses were also implemented using custom Python scripts. Because the number of MRI-derived features exceeded the number of subjects, PCA and factor analysis results were interpreted descriptively and not as stable or externally validated latent structures.

Unsupervised projection analyses were used to visualize latent MRI structure and explore whether patients grouped according to genetic or clinical phenotypes. Clinical labels were overlaid only after dimensionality reduction and were not used to construct PCA components or factors. Therefore, these projections were used only for visualization and exploratory pattern detection, not for supervised classification or prediction. Correlation analyses were then performed between imaging features or latent components and neurological manifestations.

For symptom-specific analyses, MRI-derived features were compared between patients with and without each neurological phenotype using descriptive group statistics, including means and standard deviations. The square root of the Fisher ratio was calculated as an exploratory measure of between-group separability and was used only to rank MRI-derived attributes according to their descriptive discriminative contribution. These attributes were not used as the basis for a diagnostic, prognostic, or predictive model. Therefore, no diagnostic accuracy, prognostic performance, or clinical decision threshold was derived from these analyses. All reported associations should be interpreted as uncorrected exploratory findings intended to generate hypotheses for future longitudinal and multicenter validation studies.

### 2.7. Clinical Interpretation and Network Mapping

Imaging findings were interpreted according to the strength and direction of exploratory associations, consistency with known WS neuroanatomy, and clinical plausibility. Volumetric and cortical measures were interpreted as exploratory correlates of regional atrophy or cortical thinning; ventricular volumes as indirect exploratory correlates of tissue loss; FLAIR-derived variability as an exploratory signal-based measure potentially reflecting tissue heterogeneity, gliosis, demyelination, or signal instability; and DTI measures as exploratory measures of microstructural integrity. Recurrent findings were mapped onto clinically relevant systems, including cerebello-thalamo-cortical circuits, visual relay structures, corticobulbar and brainstem pathways, posterior cortical networks, interhemispheric callosal pathways, and long-range white-matter connections.

Network labels were assigned after dimensionality-reduction analyses by reviewing the anatomical distribution of recurrent MRI-derived features and the variables with the strongest absolute loadings in each PCA- or factor-derived dimension. Because these dimensions are linear combinations of the original variables, inspection of the dominant loadings was used to identify the MRI-derived attributes contributing most strongly to each dimension. When the dominant variables showed anatomical or tissue-level coherence, a concise descriptive label was assigned to summarize the corresponding loading pattern. These labels were used as anatomical descriptors of the derived dimensions and were not used to define, constrain, or supervise the PCA or factor-analysis procedures. Furthermore, the unsupervised nature of these methods supports the exploratory value of this approach.

### 2.8. Methodological Safeguards for an Ultra-Rare Disease Cohort

The methodological framework was adapted to the constraints of an ultra-rare disease cohort, in which the availability of homogeneous MRI data is limited by both the small number of affected individuals and the need for comparable acquisition protocols. The quantitative MRI analysis was therefore restricted to standardized 3-Tesla multimodal MRI datasets with adequate image quality.

Given the high feature-to-subject ratio, with 172 MRI-derived attributes examined in 15 subjects, the corresponding phenotype-association problem is highly underdetermined. In this setting, many different combinations of imaging attributes could show similar apparent associations with neurological phenotypes. For this reason, the study was designed for exploratory pattern discovery rather than for predictive modeling, biomarker validation, or clinical classification.

Accordingly, no diagnostic, prognostic, staging, or predictive model was derived. No external validation cohort, internal resampling validation, bootstrap stability analysis, or leave-one-out validation was used to claim robustness. Although such procedures can describe internal variability, they would remain constrained by the limited information contained in the original sample and would not provide independent evidence.

The interpretation of MRI-derived associations therefore prioritized recurrence across related neurological manifestations, convergence across complementary MRI measures, anatomical and biological plausibility, and clinical interpretability. Feature-level associations, symptom-specific comparisons, dimensionality-reduction outputs, and Fisher-ratio rankings were all considered exploratory and hypothesis-generating.

The dimensionality-reduction analyses were also interpreted within this framework. With 15 MRI subjects, the rank of a centered data matrix, and consequently of the associated correlation/covariance structure, is intrinsically limited to at most 14. Therefore, the number and interpretation of principal components or factor-derived dimensions necessarily reflect the limited sampling of the patient space. With a larger sample size, the principal components, factor-derived dimensions, and relative contribution of individual MRI attributes would be expected to change as the sampled patient space becomes more representative.

Nevertheless, to assess the consistency of the exploratory findings within the available dataset, we performed a sensitivity analysis of principal component and factor-analysis configurations and evaluated the stability of the retained MRI-derived variables across filtering thresholds. The resulting MRI-derived patterns should therefore be interpreted as exploratory imaging correlates whose relative importance may change as additional harmonized MRI datasets become available. Larger multicenter, longitudinal cohorts with harmonized acquisition protocols will be required to assess reproducibility and clinical relevance.

## 3. Results

### 3.1. Principal Component Analysis

**Figure 1:**
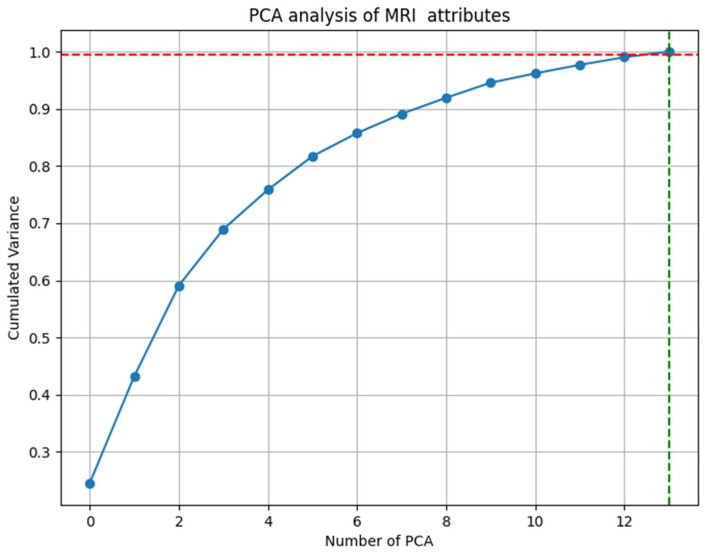
Principal component analysis of multimodal MRI-derived features. The cumulative explained variance plot shows that a small number of principal components captured a large proportion of variance across the 172 MRI-derived features. The red dashed horizontal line indicates the 99% cumulative explained variance threshold, and the green dashed vertical line indicates that 13 principal components are required to reach this threshold. Because the quantitative MRI subset included only 15 patients, this analysis was interpreted as descriptive and exploratory. PCA components were not considered stable latent structures or validated biomarkers in the absence of external replication.

Principal component analysis (PCA) was applied to the 172 MRI-derived features as a descriptive dimensionality-reduction approach to identify dominant patterns of covariation across the multimodal imaging feature space. The cumulative variance curve showed that a limited number of components captured a large proportion of the variance in the dataset. Approximately the first 10 principal components explained most of the observed variance (95%), supporting the presence of shared structure among the MRI measures. This concentration of variance is expected because the covariance matrix has rank 14, meaning that at most 14 principal components can have non-zero variance. Nevertheless, this interpretation remains strictly exploratory and is constrained by the high feature-to-subject ratio (Figure 1). Because the number of MRI-derived features exceeded the number of subjects, PCA loadings and component-level associations should not be interpreted as stable, reproducible, or externally validated latent structures.

Table 1 summarizes the strongest exploratory symptom-associated PCA components and their corresponding MRI-derived network interpretations. Each PCA network was defined descriptively by examining the original MRI measures with the highest absolute loadings within the component. These network labels were assigned after inspection of the loading structure and were intended to provide anatomical summaries of exploratory patterns, rather than predefined or independently validated neurobiological networks.

Beyond dimensionality reduction, PCA was used to explore whether specific MRI-derived components were associated with neurological symptoms. For each symptom, we identified the principal component showing the strongest absolute correlation with clinical symptom burden. The sign of the correlation was interpreted as the direction of association, whereas the magnitude of the correlation was used to estimate the strength of the symptom-component relationship.

To assign a biological interpretation to each symptom-associated component, we examined the original MRI measures with the highest absolute loadings within that PC. Measures with the strongest loadings were grouped according to their anatomical and neurobiological relevance, including cerebellar, thalamic, brainstem, visual, callosal, cortical, white-matter, and ventricular domains. This approach allowed each PC to be described as a PCA-derived MRI network rather than as an isolated mathematical component.

Correlation analyses between PCA scores and neurological symptoms identified several clinically plausible exploratory symptom–PCA component associations. PC6 showed relatively high exploratory correlations with cerebellar motor manifestations, including dysmetria and adiadochokinesia. Based on its loading profile, PC6 was interpreted descriptively as a motor–cerebellar–brainstem/callosal pattern, consistent with impaired motor coordination. PC3 was associated with gait instability, impaired tandem gait, and cognitive impairment. This component was interpreted descriptively as a global volume/ventricular–thalamic–cerebellar pattern, suggesting that gait and cognitive symptoms may reflect broader neurological involvement and distributed system-level alterations rather than isolated regional abnormalities. PC13 was associated with absent gag reflex, ataxia, and anosmia. Its loading profile suggested a thalamo–callosal–brainstem/LGN–motor pattern. Although PC13 is a higher-order component and therefore explains less global variance than earlier PCs, its association with multiple neurological manifestations suggests that higher-order components may retain regionally specific MRI information of exploratory interest. Dysphagia showed an inverse association with PC8. This component was interpreted descriptively as a brainstem–cerebellar–parietal sensory-integration pattern. The negative correlation indicates that lower PC8 scores were associated with greater dysphagia burden, but the sign should be read only as the direction of association rather than as intrinsically favorable or unfavorable.

To assess the internal stability of the PCA-derived patterns, PCA was repeated across 1000 bootstrap-resampled datasets. In each bootstrap iteration, the PCA solution was aligned with the original PCA solution, and the MRI-derived variables with the strongest absolute loadings were recorded for each principal component. Variables were then ranked according to the frequency with which they appeared among the top-loading contributors across bootstrap iterations. Table 2 summarizes these results at the component level. For each of the 14 principal components, the table lists the most stable representative MRI-derived variables, the dominant anatomical domains represented by those variables, and a descriptive MRI-derived network interpretation. The network labels were assigned descriptively from the anatomical distribution of the stable contributors and are intended to summarize the dominant MRI-derived pattern of each PCA component.

The MRI-derived variables included modality-specific quantitative attributes capturing regional signal intensity, signal heterogeneity, diffusion metrics, volumetric measures, normalized volumetric indices, and interhemispheric asymmetry. FLAIR-derived variables reflected regional signal intensity and heterogeneity, including mean intensity, 90th percentile values, standard deviation, coefficient of variation, and z-scored measures. DTI-derived variables included fractional anisotropy (FA) and mean diffusivity (MD), either as regional or normalized measures. Volumetric variables were expressed as absolute volumes in cubic millimeters or normalized to intracranial volume (ICV). Anatomical labels referred to cerebellar white matter and cortex, thalamus, lateral geniculate nucleus, brainstem, occipital white matter, corpus callosum, insular and parietal cortices, motor regions, and ventricular structures.

PCA provided a descriptive dimensionality-reduction framework to summarize these heterogeneous MRI-derived attributes into exploratory patterns with potential clinical interpretability. Cerebellar motor symptoms were mainly associated with motor–cerebellar patterns; gait and cognitive symptoms were linked to global and thalamic–cerebellar patterns; and bulbar or sensory-related manifestations were associated with brainstem, thalamic, callosal, lateral geniculate nucleus, visual, and sensory-integration patterns. These findings are compatible with a distributed model of neurological involvement in Wolfram syndrome, in which symptoms may reflect coordinated alterations across connected MRI-defined systems rather than isolated regional damage. Given the small sample size, PCA-derived associations should be considered exploratory. They suggest that both dominant and higher-order components may capture regionally specific MRI information relevant to neurological impairment in Wolfram syndrome but require longitudinal and external validation.

### 3.2. Factor Analysis

Exploratory factor analysis (FA) was applied to the 172 MRI-derived features as a complementary descriptive approach to identify plausible latent dimensions of shared covariance across the multimodal imaging feature space. FA was performed using minimum residual extraction (MINRES) with the direct oblique rotation method (Oblimin). MINRES was selected to estimate factor-derived dimensions by minimizing the residual difference between the observed correlation matrix and the factor-reproduced correlation matrix. Oblimin rotation was used because MRI-derived neuroanatomical patterns were not assumed to be fully independent, allowing factors to be correlated while improving interpretability of the loading structure.

**Figure 2:**
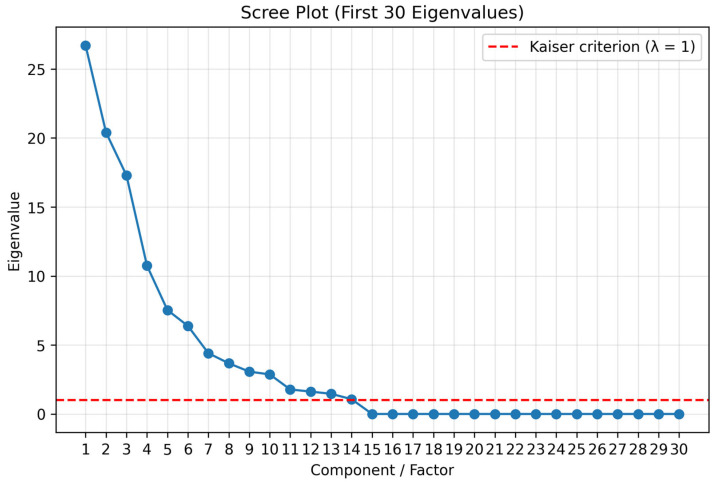
Scree plot used as a descriptive reference for exploratory factor analysis. The plot shows the first 30 eigenvalues derived from the MRI feature matrix. The dashed horizontal line indicates the Kaiser criterion threshold (lambda = 1). Because the quantitative MRI subset included only 15 patients and the number of MRI-derived features exceeded the number of subjects, factor retention was interpreted as descriptive and exploratory rather than as evidence of a stable or validated factor structure.

Factor retention was initially guided using the scree plot and the Kaiser criterion. The scree plot of the first 30 eigenvalues showed a steep decline across the first factors, followed by a progressive flattening of the curve in the higher-order factors (Figure 2). The dashed red line represents the Kaiser criterion threshold, with eigenvalues greater than 1 considered for retention as an exploratory reference. This pattern suggested that much of the shared variance was concentrated in a reduced number of latent dimensions, although this observation was interpreted only as a descriptive summary of covariance within this dataset.

To evaluate the effect of feature selection on factor-analysis adequacy, we performed a sensitivity analysis across different feature-filtering thresholds. More acceptable configurations according to conventional factor-analysis diagnostics were obtained only after substantial reduction in the MRI-derived feature set. The most informative reduced configurations retained between 3 and 10 variables, with global KMO values ranging from 0.709 to 0.812 and significant Bartlett’s tests (Table 3). In these reduced configurations, the Kaiser criterion generally suggested one or two factors.

The highest KMO values were observed in highly reduced solutions retaining 6–7 variables, with KMO values above 0.80, Bartlett’s *p*-values around 0.005, and a one-factor solution. More permissive configurations retained a larger number of variables but showed lower KMO values and a higher number of suggested factors. These findings indicate that the broader 12-factor representation should be interpreted as an exploratory decomposition used for symptom–pattern mapping, whereas the reduced sensitivity configurations provide a more constrained view of the covariance patterns that remained stable under stricter filtering.

Across the sensitivity analysis, the most frequently retained variables involved thalamic, lateral geniculate nucleus/visual pathway, brainstem–peduncular, and cerebellar attributes (Table 4). This distribution suggests anatomical convergence around a subcortical–visual relay–brainstem–cerebellar axis rather than a single isolated anatomical region. The recurrence of FLAIR, FA, MD, and volumetric measures within these regions indicates that both signal heterogeneity and microstructural integrity contributed to the retained patterns.

Several symptom–factor associations showed anatomically coherent patterns (Table 5). Motor and balance manifestations were mainly linked to cerebellar, thalamic, brainstem, occipital, and white-matter dimensions. F10 was associated with both gait instability and ataxia and was interpreted descriptively as a thalamic–brainstem–occipital/white-matter pattern related to gait, balance, and coordination. This supports the view that gait impairment and ataxia in Wolfram syndrome may reflect distributed system involvement rather than isolated cerebellar dysfunction alone.

F12 was associated with dysmetria and was interpreted as a cerebellar–cortical sensory-integration pattern, consistent with the classical link between dysmetria and cerebellar dysfunction while also suggesting a contribution from cortical and sensory-integration regions. F5 was associated with cognitive impairment and adiadochokinesia and was interpreted as a global structural/disconnection pattern, suggesting that cognitive impairment and motor sequencing deficits may reflect broader system-level involvement.

Bulbar-related symptoms were associated with distinct but anatomically related factor-derived patterns. Dysphagia was most strongly associated with F7, interpreted descriptively as a sensory–motor swallowing pattern. Sialorrhea was associated with F2, suggesting involvement of cerebellar–thalamic–white-matter circuits relevant to oral motor control. Absent gag reflex was associated with F9, consistent with a broader callosal–ventricular or global burden pattern affecting bulbar-related signs.

The internal stability of the factor-derived patterns was assessed using 1000 bootstrap-resampled datasets. The most frequently retained top-loading variables for each factor are summarized in Table 6. Bootstrap-stable contributors involved cerebellar white matter and cortex, thalamic and lateral geniculate nucleus regions, brainstem–pontine structures, occipital white matter, corpus callosum, and global volumetric measures. These findings support the internal consistency of the main exploratory anatomical patterns, particularly those involving cerebellar–brainstem, thalamic–visual relay, and global structural burden dimensions.

The factor-analysis results identified exploratory MRI-derived anatomical patterns associated with neurological symptoms. Motor and balance manifestations were mainly linked to cerebellar, thalamic, brainstem, visual relay, and white-matter dimensions; cognitive impairment was associated with a global structural/disconnection pattern; and bulbar symptoms were associated with sensory–motor, oral–motor, and broader network-burden patterns. Together, these findings support a distributed model of neurological involvement in Wolfram syndrome, in which symptoms may reflect coordinated alterations across connected MRI-defined systems rather than isolated regional abnormalities.

The anatomical interpretation of PCA components and factors was based on the MRI-derived measures with the highest absolute loadings. The resulting PCA-derived and factor-derived descriptive pattern labels are provided in Supplementary Tables S1 and S2, respectively. Overall, PCA and factor-analysis results showed broadly convergent exploratory symptom–pattern associations, with recurrent involvement of cerebellar, thalamic, brainstem, visual relay, white-matter, and broader system-level patterns.

In the absence of an external validation cohort, these findings should be interpreted as descriptive, hypothesis-generating MRI-derived summaries rather than as validated latent factors, reproducible biological networks, or clinically applicable biomarkers.

### 3.3. Unsupervised Projection Analysis

The MRI-derived feature matrix was projected onto the first two principal components and the first two latent factors to visualize the global structure of the data, explore apparent clustering patterns, and explore whether patients showed visual separation according to different phenotypes: WFS1-related mutation, dysphagia, ataxia, gait instability, and cognitive impairment. These projections were generated using unsupervised dimensionality reduction. Phenotype labels were not used to construct the PCA components or latent factors; they were overlaid afterward only for visualization and exploratory interpretation.

**Figure 3:**
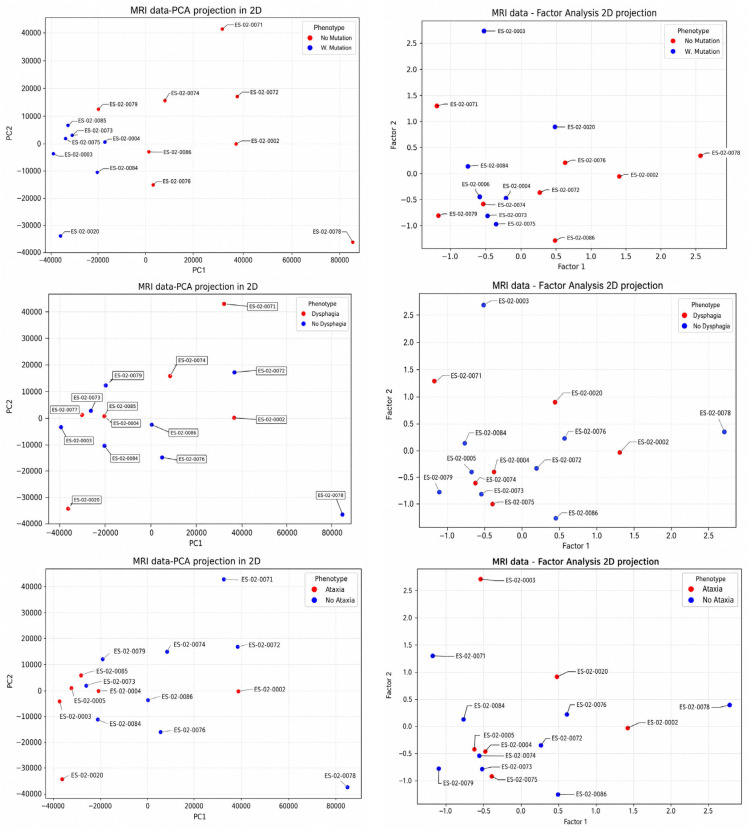

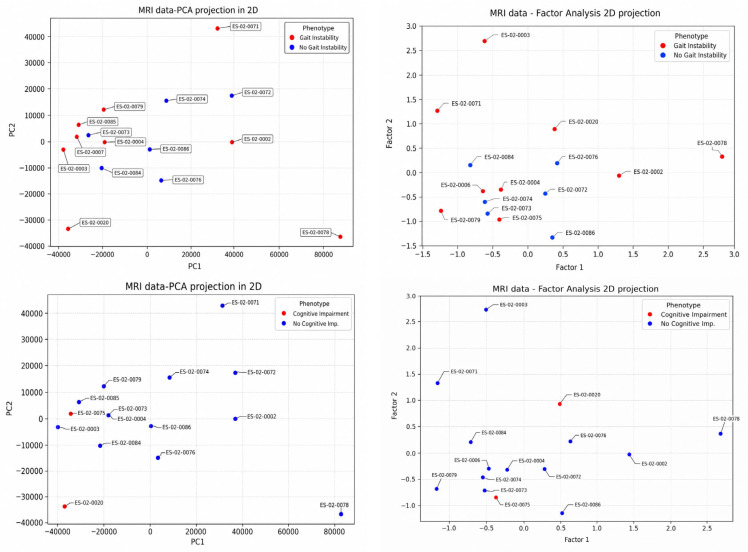
PCA and factor-analysis projections of MRI-derived features by phenotype. The first two principal components and factor-derived dimensions were used to visualize group separation according to WFS1-related mutation status, dysphagia, ataxia, gait instability, and cognitive impairment. PCA showed visual separation for WFS1-related mutation status, ataxia, and cognitive impairment, whereas dysphagia showed partial overlap in both methods. Gait instability showed a pattern similar to ataxia.

Figure 3 shows the results of the unsupervised projection analysis. The projections indicate that the apparent visual separation of clinical or genetic groups varied according to the phenotype examined. These projections were generated using unsupervised dimensionality-reduction methods, with phenotype labels overlaid only after projection; therefore, they should not be interpreted as supervised classification, prediction, or validated group discrimination. Linear separability refers to the possibility of separating groups by a straight line, or by a hyperplane in higher-dimensional spaces, within the projected feature space. Depending on the phenotype, different degrees of sample mixing were observed between groups.

For WFS1-related mutation status, PCA showed more apparent visual separation than factor analysis, suggesting that dominant axes of MRI variability could be related to genetic background. This finding must be interpreted cautiously because PCA is unsupervised, the imaging subset is small, and no externally validated supervised classifier was trained. The apparent visual separation observed for WFS1-related mutation status should therefore not be interpreted as evidence of reliable prediction. Rather, it suggests that genetic background could be explored further in relation to the multimodal MRI feature space and warrants confirmation in larger cohorts with prespecified genotype categories, harmonized MRI protocols, and independent validation.

These results suggest that PCA may be useful for visualizing global MRI variability and broader genetic or clinical phenotypes, whereas factor analysis may provide complementary descriptive insight into more specific latent symptom-related patterns.

### 3.4. Symptom-Associated Correlation Analysis

Table 7 highlights the MRI-derived features showing the most recurrent exploratory associations with neurological phenotypes in Wolfram syndrome and summarizes their descriptive association patterns. For each imaging metric, three complementary indicators were reported: the number of neurological manifestations associated with the feature, the mean absolute correlation across associated symptoms, and the maximum absolute correlation observed between the MRI feature and an individual phenotype. From a clinical standpoint, features linked to several manifestations may represent exploratory cross-domain imaging correlates of neurological burden, whereas features with high maximum correlations may capture possible symptom-specific vulnerability.

Diffusion-based metrics also appeared prominently in Table 7. Fractional anisotropy and mean diffusivity measures in the cerebellar cortex, cerebellar white matter, brainstem, thalamus, and lateral geniculate nucleus showed recurrent exploratory associations with multiple symptoms. These findings are compatible with a possible contribution of microstructural alterations in cerebellar, brainstem, thalamic, and visual pathway regions to the clinical expression of the disease. Cortical measures were also represented among the recurrently associated features, particularly occipital cortical thickness, with the right occipital cortex associated with the largest number of neurological manifestations.

The correlation analysis pointed to a distributed pattern of structural and microstructural alterations rather than to a single region-specific imaging correlate. The most recurrently associated MRI features involved the thalamus, lateral geniculate nucleus, cerebellum, brainstem, white matter, ventricular system, and occipital cortex. Thalamic and LGN FLAIR-derived metrics showed recurrent associations, suggesting possible involvement of relay and integration nuclei, whereas diffusion alterations in the cerebellum, brainstem, and white matter may reflect reduced microstructural integrity. Ventricular enlargement may reflect global brain atrophy or broader tissue loss, although longitudinal data are required to determine progression.

In this context, the pattern summarized in Table 7 is compatible with a multimodal representation of CNS involvement in Wolfram syndrome, involving FLAIR signal heterogeneity, diffusion abnormalities, regional volumetric changes, ventricular enlargement, and cortical thickness alterations across cerebello-thalamo-cortical circuits, visual relay structures, white matter pathways, and the brainstem.

Beyond the ranking of the most recurrent MRI features, we also examined the strongest individual structure-symptom correlations to identify possible symptom-specific neuroimaging patterns. Table 8 summarizes the main results of this exploratory analysis.

Coordination and gait-related symptoms showed recurrent exploratory associations with cerebellar, thalamic, brainstem, and white matter measures. Ataxia was associated with FLAIR-derived metrics in the lateral geniculate nucleus and thalamus, as well as with cerebellar and parietal measures. Gait and tandem gait impairment were mainly related to brainstem, cerebellar cortex, and white matter integrity metrics, suggesting possible involvement of motor coordination and balance systems.

Bulbar and sensory symptoms showed a partly distinct exploratory pattern. Dysphagia was negatively associated with cortical measures in parietal, insular, motor, and occipital regions, suggesting that reduced cortical thickness or structural integrity in these areas could be related to swallowing impairment. Dysmetria was associated with cerebellar white matter and lateral geniculate nucleus metrics, while anosmia showed associations with cerebellar cortical measures. Additional findings included associations between hyperreflexia and corpus callosum or parietal cortical measures.

The direction of the correlations also provided important insights. Positive associations involving FLAIR signal features suggest that increased signal abnormality or heterogeneity may reflect greater tissue involvement. Conversely, negative correlations involving cortical thickness or fractional anisotropy indicate that lower structural or microstructural integrity may be associated with greater clinical involvement. These structure-symptom associations are compatible with distributed MRI-derived exploratory imaging patterns involving cerebellar, thalamic, brainstem, white matter, cortical, and visual relay structures in Wolfram syndrome.

Clinically, these correlations suggest that quantitative MRI should be interpreted as an adjunct to neurological examination rather than as a standalone diagnostic tool. The convergence of thalamic/LGN FLAIR heterogeneity, cerebellar and brainstem diffusion abnormalities, ventricular enlargement, cortical thinning, and corpus callosum involvement may provide new insights into neurological burden, support the exploratory identification of patients who may benefit from closer swallowing, gait, or cognitive follow-up, and inform the selection of candidate quantitative endpoints for future longitudinal studies.

### 3.5. Symptom-Specific Alteration and Network Inference

For the symptom-specific exploratory analyses, each MRI-derived feature was compared between patients with and without the corresponding clinical phenotype, including dysphagia, ataxia/gait instability, and cognitive impairment. For each feature, group means and standard deviations were computed, and the square root of the Fisher ratio was used as an exploratory measure of group separability, with higher values indicating greater descriptive separation between patients with and without the corresponding phenotype. The direction of change was defined according to whether the feature was increased or decreased in the symptomatic group.

#### 3.5.1. Dysphagia

Table 9 and Table 10 summarize the dysphagia-specific MRI analysis, first identifying the individual MRI features with the greatest exploratory group-separation values between patients with and without dysphagia and then grouping these alterations into broader exploratory imaging-feature categories for biological and clinical interpretation.

From a clinical perspective, the dysphagia-specific MRI profile is clinically relevant because swallowing impairment may indicate bulbar or corticobulbar involvement. The features with the highest group-separation values were the reduced cortical thickness in parietal, insular, motor, and occipital regions; lower thalamic, brainstem, and pontine volumes; reduced mid-posterior corpus callosum volume; lower cerebellar white-matter fractional anisotropy; and decreased parietal and motor gray-matter volumes. These findings suggest that dysphagia in Wolfram syndrome may not reflect an isolated cranial-nerve sign alone, but could be associated with distributed corticobulbar, cerebello-thalamo-cortical, brainstem, and long-range connectivity involvement.

#### 3.5.2. Ataxia and Gait Instability

Table 11, Table 12, Table 13 and Table 14 summarize the ataxia- and gait-instability-specific MRI analyses.

The ataxia and gait-instability analyses showed a highly overlapping exploratory MRI-derived profile, supporting the clinical impression that both phenotypes share common neuroanatomical substrates. In both cases, the features with the highest descriptive group-separation values included reduced cortical thickness in parietal, insular, and occipital regions, increased FLAIR heterogeneity in the thalamus and lateral geniculate nucleus, altered LGN/thalamus ratios, reduced thalamic volumes, and ventricular enlargement. This common pattern supports the involvement of cerebello-thalamo-cortical circuits, deep gray-matter relay nuclei, and posterior cortical regions in motor coordination and balance impairment.

Despite this overlap, the phenotypes were not identical. The ataxia profile was more strongly characterized by thalamic volume loss, parietal asymmetry, and parietal gray-matter reduction, suggesting a greater contribution of sensory–motor integration and cortical network organization. Gait instability showed a more prominent contribution of white-matter integrity measures, including reduced cerebellar and global white-matter FA, as well as posterior white-matter FLAIR signal alterations. This suggests that gait instability may depend more heavily on microstructural disconnection across distributed white-matter pathways.

Clinically, ataxia and gait instability should therefore be considered related but partially separable expressions of a shared network-level involvement. Ataxia may be more closely linked to thalamo-cortical and parietal integration abnormalities, whereas gait instability may additionally reflect cerebellar and global white-matter disconnection.

#### 3.5.3. Cognitive Impairment

Table 15 and Table 16 summarize the cognitive-impairment-specific MRI analysis, first by identifying the MRI features with the greatest exploratory discriminative power between patients with and without cognitive impairment, and then by grouping these findings into broader exploratory imaging-feature categories for biological interpretation.

The cognitive-impairment profile suggests that cognitive symptoms in Wolfram syndrome may arise from combined cortical involvement and disruption of long-range subcortical, cerebellar, callosal, and ventricular systems. The involvement of insular, motor, parietal, thalamic, brainstem, pontine, and cerebellar white-matter features supports the interpretation of cognitive impairment as a clinical manifestation associated with broader network-level involvement rather than a purely cortical phenomenon.

#### 3.5.4. Exploratory MRI-Derived Feature Patterns According to Neurological Burden

To explore whether multimodal MRI-derived features were associated with neurological burden, MRI-derived attributes were organized into descriptive feature categories based on the symptom-specific comparisons and on the anatomical interpretation of the most recurrent MRI patterns. Because this study is cross-sectional, these categories should not be interpreted as temporal disease stages, progression markers, or validated biomarkers. The results are summarized in Table 17 and Table 18.

Table 17 organizes MRI-derived feature categories according to their apparent cross-sectional association with neurological burden. Deep gray-matter FLAIR heterogeneity, particularly involving the thalamus and lateral geniculate nucleus, together with ventricular enlargement, was interpreted as a signal-based and structural pattern potentially reflecting tissue heterogeneity, gliosis, demyelination, microstructural stress, or secondary tissue loss. Cortical thinning in parietal, insular, and occipital cortices, together with white-matter integrity measures and thalamic volume loss, suggested involvement of cortical integration networks, cerebellar and long-range white-matter pathways, and sensory–motor relay hubs. Structural alterations involving motor cortical regions, brainstem and pons, mid-posterior corpus callosum, and cerebellar white matter were associated with greater neurological burden and may reflect broader involvement of motor execution, bulbar, interhemispheric, and cerebellar output systems.

Table 18 further organizes these findings into two major MRI-derived feature domains: structural alterations and signal-based tissue alterations. Structural alterations included cortical thinning, gray-matter volume loss, thalamic volume reduction, brainstem and pontine volume reduction, corpus callosum reduction, and cerebellar white-matter involvement. Signal-based tissue alterations included FLAIR heterogeneity, diffusion variability, ventricular enlargement, and posterior white-matter FLAIR signal abnormalities. These domains should be interpreted as complementary exploratory MRI-derived patterns associated with neurological burden, rather than as validated markers of disease progression.

These cross-sectional findings suggest that neurological burden in Wolfram syndrome is not explained by a single MRI-derived attribute, but by the convergence of multiple structural, diffusion-based, and signal-based abnormalities. Greater neurological burden was associated with distributed cortical, thalamic, brainstem, callosal, and cerebellar involvement, whereas FLAIR heterogeneity, diffusion variability, ventricular enlargement, and posterior white-matter signal abnormalities provided complementary information about tissue heterogeneity and network-level involvement. These findings remain exploratory and require longitudinal validation before any temporal, prognostic, or clinical-monitoring use can be supported.

## 4. Discussion

### 4.1. Main Clinical Findings

This exploratory study describes a distributed multimodal MRI-derived pattern associated with neurological manifestations in Wolfram syndrome. The most informative findings were not confined to a single region, but involved cortical thickness, thalamic and lateral geniculate nucleus signal heterogeneity, brainstem and pontine volume, cerebellar white-matter integrity, ventricular enlargement, corpus callosum volume, and posterior cortical measures. This is consistent with a network-level model of WS affecting cerebello-thalamo-cortical circuits, visual relay structures, corticobulbar and brainstem pathways, and long-range white-matter connections [14,15,16,17,18,19,20].

The exploratory relevance of this multimodal approach lies in its potential to relate MRI-derived alterations to functional neurological phenotypes in future studies. Dysphagia, ataxia, gait instability, and cognitive impairment showed partially overlapping but distinguishable MRI-derived patterns. This suggests that quantitative MRI could support future efforts to characterize neurological burden more granularly than a binary affected/unaffected description.

### 4.2. Deep Gray Matter and Visual Relay Structures as Recurrent Exploratory Imaging Correlates

FLAIR-derived heterogeneity in the thalamus and lateral geniculate nucleus emerged as one of the most recurrent findings across clinical domains. The thalamus is a major sensory–motor and cognitive relay hub, while the lateral geniculate nucleus is central to visual pathway integrity. In WS, where optic atrophy, posterior pathway involvement, and multisystem neurological progression coexist, signal heterogeneity in these deep relay structures may provide exploratory information beyond simple volume measurement.

Increased coefficient of variation or standard deviation on FLAIR should be interpreted cautiously because these measures are sensitive to acquisition and preprocessing differences. Nevertheless, the recurrent association of thalamic and LGN signal heterogeneity with multiple neurological phenotypes suggests that tissue instability, gliosis, demyelination, or microstructural stress in deep gray matter may represent a recurrent exploratory imaging correlate of neurological involvement.

### 4.3. Dysphagia and Bulbar Network Involvement

Dysphagia was associated with a broad exploratory MRI-derived pattern that included cortical thinning, thalamic volume reduction, brainstem and pontine volume reduction, reduced corpus callosum volume, lower cerebellar white-matter FA, ventricular enlargement, and increased FLAIR heterogeneity. Clinically, this pattern suggests that swallowing impairment in WS is likely to reflect distributed corticobulbar, cerebellar, thalamic, and brainstem involvement rather than isolated local dysfunction.

This interpretation may be relevant for future patient-management research. Dysphagia can be associated with aspiration, nutritional compromise, respiratory complications, and reduced quality of life. In future longitudinal studies, quantitative MRI-derived patterns linked to dysphagia could be evaluated alongside swallowing assessment, speech and language therapy needs, nutritional evaluation, and respiratory risk monitoring.

### 4.4. Ataxia, Gait Instability, and Cerebello-Thalamo-Cortical Disconnection

Ataxia and gait instability showed a shared pattern involving cortical thinning, thalamic and LGN FLAIR heterogeneity, altered thalamic/LGN ratios, thalamic volume loss, ventricular enlargement, and cerebellar or global white-matter integrity abnormalities. This is consistent with prior evidence that WS affects brainstem, cerebellar, thalamic, and white-matter systems rather than a single anatomical compartment [14,15,16,17,18,19,20].

The partial dissociation between ataxia and gait instability may be clinically relevant. Ataxia appeared more closely linked to thalamo-cortical and parietal integration measures, whereas gait instability showed a greater contribution from cerebellar and global white-matter disconnection. This suggests that detailed MRI phenotyping could be evaluated in future longitudinal studies to help distinguish coordination-dominant impairment from balance and locomotor-network impairment.

### 4.5. Cognitive Involvement and Distributed Network Vulnerability

Cognitive impairment was associated with cortical, subcortical, cerebellar, callosal, brainstem, and ventricular abnormalities. This supports the view that cognitive symptoms in WS may reflect distributed network vulnerability rather than isolated cortical degeneration. The involvement of the thalamus, corpus callosum, cerebellar white matter, ventricular system, and posterior cortical regions is compatible with disruption of long-range connectivity and integrative networks.

For future clinical research, these findings support evaluating systematic neuropsychological screening alongside MRI patterns showing combined cortical thinning, callosal reduction, ventricular enlargement, and cerebellar or thalamic abnormalities. They also suggest that cognitive status should be considered as a relevant exploratory clinical endpoint in future imaging studies and therapeutic trials.

### 4.6. Research Implications and Future Trial Design

The results support the use of multimodal MRI as a quantitative adjunct to clinical examination in WS. Exploratory MRI-derived patterns may help define candidate imaging endpoints for future longitudinal and interventional studies, but they cannot currently be used for diagnosis, prognosis, clinical monitoring, or treatment selection. This is particularly important in ultra-rare diseases, where clinical trials are limited by small sample sizes and where sensitive outcome measures are needed [13,22,23,24,25].

A future imaging-endpoint framework should not rely on a single metric. Instead, a composite approach combining deep gray-matter FLAIR heterogeneity, cortical thickness, thalamic and brainstem volumes, cerebellar and global white-matter integrity, ventricular size, and callosal volume may provide a more comprehensive basis for future evaluation. Such a framework could be evaluated in future studies to explore whether combined MRI-derived features are associated with bulbar dysfunction, motor coordination impairment, gait instability, and cognitive involvement.

### 4.7. Strengths and Limitations

The main strength of this study is the integration of multiple MRI domains in a genetically confirmed Wolfram syndrome cohort with clinically relevant neurological phenotypes. The analysis was deliberately designed to prioritize interpretability, anatomical plausibility, and consistency across MRI-derived attributes, which is appropriate for an ultra-rare disorder with limited sample availability.

The most important limitation is the small homogeneous imaging subset of 15 patients. Because the number of MRI-derived features exceeded the number of subjects, the phenotype-association problem is highly underdetermined, increasing the risk of unstable correlations and overfitting. Although dimensionality reduction, stability analyses of MRI-derived attributes, and clinically guided interpretation were used to mitigate this risk, all findings should be considered exploratory. The relative contribution of individual features, principal components, and factor-derived dimensions may change as additional patients are included, and the sampled patient space becomes more representative.

This consideration is particularly relevant in Wolfram syndrome, where neurological manifestations are heterogeneous and phenotype-specific projections may show different degrees of overlap or sample mixing. Therefore, the observed patterns should be interpreted as internally coherent exploratory imaging correlates rather than as fixed neurobiological networks, validated biomarkers, or clinically applicable signatures.

Additional limitations include the absence of an independent validation cohort, potential scanner-model and protocol effects, incomplete documentation of sequence-level acquisition parameters, limited longitudinal imaging, and binary coding of complex neurological phenotypes. Future multicenter studies should use harmonized MRI acquisition protocols and report acquisition parameters, software versions, preprocessing settings, and quality-control procedures in sufficient detail to support reproducibility and determine which MRI-derived attributes remain stable across broader and more representative Wolfram syndrome populations.

### 4.8. Future Directions

Future studies should evaluate these exploratory MRI-derived features in larger multicenter cohorts using harmonized MRI protocols, standardized neurological scales, swallowing assessments, neuropsychological testing, and longitudinal follow-up. External validation should assess whether the proposed MRI-derived patterns are associated with clinical progression, functional decline, treatment response, or relevant patient outcomes. Integration with genotype, ophthalmological measures, hearing outcomes, endocrine status, plasma biomarkers, and patient-reported outcomes may improve future neurological characterization and exploratory patient stratification.

## 5. Conclusions

This study supports an exploratory multimodal MRI-derived framework for the neurological characterization of Wolfram syndrome. The most relevant alterations involved cortical thinning, thalamic, brainstem, and pontine volume reduction, cerebellar and white-matter microstructural alterations, FLAIR signal heterogeneity in deep nuclei and the lateral geniculate nucleus, ventricular enlargement, corpus callosum involvement, and posterior cortical abnormalities.

Greater neurological involvement was associated with distributed structural alterations involving cortical, thalamic, brainstem, pontine, callosal, and cerebellar systems. In contrast, deep gray-matter FLAIR heterogeneity, diffusion variability, and ventricular enlargement may represent complementary exploratory imaging correlates of tissue heterogeneity and network-level involvement. Dysphagia, ataxia, gait instability, and cognitive impairment were associated with partially overlapping but clinically distinguishable MRI-derived patterns, supporting a distributed network-level model of central nervous system involvement in Wolfram syndrome.

Although several MRI-derived attributes showed recurrent and internally stable contributions, these findings remain exploratory and may change as larger and more representative samples become available. Therefore, the observed patterns should be interpreted as exploratory MRI-derived features for future evaluation in neurological characterization and longitudinal research, rather than as validated biomarkers. Future longitudinal, multicenter studies with harmonized MRI acquisition protocols are required to assess their reproducibility, clinical relevance, and potential relevance for future longitudinal assessment.

## Figures and Tables

**Table 1:** Exploratory associations between PCA-derived MRI patterns and neurological manifestations in Wolfram syndrome. For each neurological manifestation, the table shows the principal component with the strongest exploratory correlation, the correlation coefficient, anatomical plausibility, and a descriptive interpretation based on the strongest PCA loadings. PC = principal component; LGN = lateral geniculate nucleus. In the absence of external validation, these correlations should be interpreted as exploratory.

Neurological Manifestation	Strongest Associated PC	Correlation Coefficient	Anatomical Plausibility	Descriptive Anatomical Interpretation Based on Strongest PCA Loadings
Dysphagia	PC8	−0.62	Plausible	Brainstem–cerebellar–parietal sensory-integration pattern
Sialorrhea	PC2	0.54	Partly plausible	Thalamic–cerebellar–occipital white-matter diffusion pattern
Absent gag reflex	PC13	0.53	Partly plausible	Thalamic–callosal–brainstem/LGN-motor pattern
Dysmetria	PC6	0.63	Highly plausible	Motor–cerebellar–brainstem/callosal pattern
Gait instability	PC3	0.48	Plausible	Global volume/ventricular–thalamic–cerebellar pattern
Ataxia	PC13	0.45	Plausible	Thalamic–callosal–brainstem/LGN–motor pattern
Cognitive impairment	PC3	0.44	Highly plausible	Global volume/ventricular–thalamic–cerebellar pattern
Anosmia	PC13	0.48	Less direct	Thalamic–callosal–brainstem/LGN-motor pattern
Impaired tandem gait	PC3	0.46	Plausible	Global volume/ventricular–thalamic–cerebellar pattern
Dysarthria	PC4	0.53	Partly plausible	Visual–motor asymmetry and FLAIR pattern
Adiadochokinesia	PC6	0.60	Highly plausible	Motor–cerebellar–brainstem/callosal pattern

**Table 2:** Bootstrap-stable PCA-derived MRI patterns and descriptive network interpretation. Variables represent the most frequently retained top-loading contributors across bootstrap PCA resamples. Network interpretations are descriptive anatomical summaries of PCA-derived patterns and should not be interpreted as validated neurobiological networks. In the absence of external validation in larger populations, these attributes and their correlation with the main PCA components should be interpreted as exploratory. [Abbreviations: FA, fractional anisotropy; MD, mean diffusivity; FLAIR, fluid-attenuated inversion recovery; Cereb, cerebellum; WM, white matter; Ctx, cortex; Thal, thalamus; LGN, lateral geniculate nucleus; CC, corpus callosum; ICV, intracranial volume; L/R, left/right; p90, 90th percentile; std, standard deviation; cv, coefficient of variation; normWM, normalized to white matter; LR_asym, left–right asymmetry index; mm3, cubic millimeters].

PCA Component	Most Stable MRI-Derived Attributes	Dominant Anatomical Domains	Descriptive MRI-Derived Network Interpretation
PC1	FLAIR_Cereb_WM_R_p90; FLAIR_Cereb_WM_R_mean; FLAIR_Cereb_WM_R; FLAIR_Cereb_WM_L_p90; FLAIR_Cereb_Ctx_L_p90; FLAIR_Brainstem_p90	Cerebellar white matter/cortex, brainstem, posterior white matter	Cerebellar–brainstem FLAIR signal-heterogeneity pattern
PC2	MD_Cereb_WM_L_normWM; LGN_Thal_ratio_R; Cereb_WM_R_mm3; MD_Cereb_Ctx_R_normWM; LGN_Thal_ratio_L; Cereb_WM_L_mm3	Cerebellum, thalamus/LGN, pons/peduncle	Cerebellar–thalamic–visual relay/brainstem structural-diffusion pattern
PC3	Insula_ICV; Parietal_ICV; Cereb_ICV; Motor_ICV; Thal_ICV; Brainstem_ICV	Cortical, cerebellar, thalamic, and brainstem regions	Global cortical–subcortical–brainstem burden pattern
PC4	FLAIR_occWM_z; FA_Cereb_WM_R; FA_Cereb_WM_R_normWM; FA_Brainstem_std; MD_Cereb_WM_R_std; MD_Cereb_WM_L_std	Occipital white matter, cerebellar white matter, brainstem	Occipital–cerebellar–brainstem white-matter integrity/signal pattern
PC5	MD_Cereb_WM_R_std; MD_Cereb_Ctx_R_std; MD_Cereb_WM_L_std; FA_LGN_R; MD_Brainstem_std; MD_Cereb_Ctx_L_std	Cerebellum, LGN, brainstem, ventricular system	Cerebellar diffusion heterogeneity with visual relay and brainstem involvement
PC6	FLAIR_Cereb_WM_L_cv; FLAIR_Cereb_WM_R_cv; CC_Anterior_mm3; FA_occWM_mean; FA_Brainstem; Motor_LR_asym	Cerebellum, corpus callosum, occipital white matter, brainstem	Cerebellar signal-heterogeneity and callosal/occipital–brainstem connectivity pattern
PC7	FLAIR_Brainstem_cv; Insula_LR_asym; MD_LGN_R_std; Thalamus_LR_asym; Motor_LR_asym; FA_Brainstem	Brainstem, LGN, thalamus, insular/motor asymmetry	Brainstem–visual relay–thalamic asymmetry pattern
PC8	Insula_LR_asym; Insula_R_grayvol_mm3; FA_Brainstem; Brainstem_deg_index; Parietal_LR_asym; CC_Mid_Anterior_mm3	Insula, brainstem, parietal cortex, corpus callosum	Insular–brainstem–callosal asymmetry/burden pattern
PC9	Cereb_Ctx_L_mm3; Thal_L_mm3; ICV_mm3; Cereb_Ctx_R_mm3; Brainstem_mm3; Ventricle_3_mm3	Cerebellar cortex, thalamus, brainstem, ventricular system	Subcortical–brainstem–cerebellar volumetric burden pattern
PC10	ICV_mm3; Thal_L_mm3; Thal_R_mm3; Cereb_Ctx_L_mm3; Brainstem_mm3; Cereb_Ctx_R_mm3	ICV, thalamus, cerebellar cortex, brainstem	Global volumetric axis dominated by thalamic–cerebellar–brainstem volumes
PC11	ICV_mm3; Thal_L_mm3; Thal_R_mm3; Brainstem_mm3; Cereb_Ctx_L_mm3; Cereb_Ctx_R_mm3	ICV, thalamus, brainstem, cerebellum	Global structural burden pattern with thalamic–brainstem–cerebellar dominance
PC12	ICV_mm3; Thal_L_mm3; Brainstem_mm3; Thal_R_mm3; Cereb_Ctx_L_mm3; Cereb_Ctx_R_mm3	ICV, thalamus, brainstem, cerebellum	Global thalamic–brainstem–cerebellar volume pattern
PC13	Thal_L_mm3; Brainstem_mm3; ICV_mm3; Thal_R_mm3; Cereb_Ctx_L_mm3; Cereb_Ctx_R_mm3	Thalamus, brainstem, ICV, cerebellum	Thalamic–brainstem–cerebellar volumetric axis
PC14	Thal_L_mm3; Brainstem_mm3; ICV_mm3; Thal_R_mm3; Cereb_Ctx_L_mm3; Cereb_Ctx_R_mm3	Thalamus, brainstem, ICV, cerebellum	Thalamic–brainstem–cerebellar/global volumetric burden pattern

**Table 3:** Sensitivity analysis of reduced factor-analysis configurations. This table shows the most informative feature-filtering configurations obtained by varying the KMO, minimum-correlation, and maximum-correlation thresholds before exploratory factor analysis. For each configuration, the number of retained MRI-derived variables, retained variables per subject, global KMO value, Bartlett’s test *p*-value, and number of Kaiser-suggested factors are reported. The most adequate diagnostic profiles were observed only after substantial feature reduction, supporting the interpretation of these factor-analysis outputs as compact descriptive summaries rather than stable latent factor structures. In the absence of external validation in larger populations, these results should be considered exploratory.

KMO Threshold	Minimum Correlation	Maximum Correlation	Retained Variables	Variables per Subject	Global KMO	Bartlett *p*-Value	Kaiser Factors	Interpretation
0.45	0.35	0.75	7	0.47	0.812	0.00543	1	Highest KMO; compact one-factor descriptive solution
0.50	0.35	0.75	6	0.40	0.809	0.00521	1	Highly similar compact one-factor solution
0.45	0.40	0.75	4	0.27	0.771	0.00698	1	Very parsimonious one-factor solution
0.60	0.40	0.80	10	0.67	0.761	8.06 × 10^−6^	2	Acceptable but less parsimonious two-factor solution
0.40	0.35	0.75	8	0.53	0.733	0.00632	2	Reduced exploratory two-factor solution
0.50	0.40	0.75	3	0.20	0.709	0.00312	1	Highly parsimonious but very reduced solution

**Table 4:** Stability of MRI-derived variables across feature-filtering configurations. The table shows the MRI-derived variables most consistently retained during the factor-analysis sensitivity exploration. “Configurations retained” indicates the number of filtering configurations in which each variable was retained out of 125 tested configurations, and “Frequency (%)” indicates the corresponding percentage. The most stable variables converged on thalamic, lateral geniculate nucleus/visual pathway, brainstem–peduncular, and cerebellar domains, supporting a recurrent subcortical–visual relay–brainstem–cerebellar MRI-derived pattern across filtering thresholds. In the absence of external validation in larger populations, these results should be considered exploratory. Abbreviations: FA, fractional anisotropy; MD, mean diffusivity; FLAIR, fluid-attenuated inversion recovery; Thal, thalamus; LGN, lateral geniculate nucleus; Cereb, cerebellum; WM, white matter; Ctx, cortex; L/R, left/right; std, standard deviation; normWM, normalized to white matter; mm3, cubic millimeters.

Attribute	Configurations Retained	Frequency (%)	Main Anatomical Domains	Imaging Attribute
FA_Thal_R	125	100.0	Thalamus	FA
FLAIR_Thal_L	125	100.0	Thalamus	FLAIR
peduncle_vol_mm3	120	96.0	Brainstem/peduncle	Volume
FA_LGN_R	117	93.6	Lateral geniculate nucleus/visual pathway	FA
FA_Thal_L_std	117	93.6	Thalamus	FA
MD_Thal_L	115	92.0	Thalamus	MD
MD_Brainstem	114	91.2	Brainstem	MD
FA_Cereb_WM_L	112	89.6	Cerebellar white matter	FA
FLAIR_Cereb_Ctx_L_std	100	80.0	Cerebellar cortex	FLAIR
FLAIR_Thal_R_std	100	80.0	Thalamus	FLAIR
FLAIR_Thal_L_std	100	80.0	Thalamus	FLAIR
FA_Thal_L_normWM	100	80.0	Thalamus	FA
MD_LGN_R	100	80.0	Lateral geniculate nucleus/visual pathway	MD
FLAIR_LGN_R_std	100	80.0	Lateral geniculate nucleus/visual pathway	FLAIR

**Table 5:** Exploratory associations between factor-derived MRI patterns and neurological symptoms in Wolfram syndrome. The table summarizes, for each neurological symptom, the factor showing the strongest exploratory correlation, the corresponding uncorrected correlation coefficient, the anatomical rationale for the association, and the descriptive MRI-derived pattern interpretation based on the strongest factor loadings. Factor scores were interpreted descriptively according to their dominant MRI-derived loadings and were used for exploratory symptom–pattern mapping. In the absence of external validation in larger populations, these associations should be considered exploratory and should not be interpreted as validated factor solutions, biomarkers, or diagnostic, prognostic, or predictive models.

Neurological Symptom	Strongest Associated Exploratory Factor	Uncorrected Correlation	Anatomical Rationale	Descriptive MRI-Derived Pattern Interpretation
Dysphagia	F7	0.65	Bulbar/sensory–motor involvement	Sensory–motor swallowing pattern
Sialorrhea	F2	−0.50	Oral motor control pathways	Cerebellar–thalamic–white-matter oral motor pattern
Absent gag reflex	F9	−0.46	Bulbar-related network burden	Callosal–ventricular/global burden pattern affecting bulbar-related signs
Dysmetria	F12	0.50	Cerebellar and sensory-integration involvement	Cerebellar–cortical sensory-integration pattern
Gait instability	F10	0.59	Gait, balance, and long-tract involvement	Thalamic–brainstem–occipital/white-matter gait and balance pattern
Ataxia	F10	0.60	Coordination and balance pathways	Cerebellar–thalamic–brainstem coordination pattern
Cognitive impairment	F5	0.65	Global atrophy and disconnection	Global structural/disconnection pattern
Anosmia	F4	0.35	Indirect or less specific anatomical relationship	Thalamic motor/structural involvement pattern
Impaired tandem gait	F6	0.44	Fine balance and coordination pathways	Brainstem–cerebellar–thalamic fine-balance pattern
Dysarthria	F3	0.39	Motor speech pathway involvement	Brainstem/motor speech pathway pattern
Adiadochokinesia	F5	0.51	Motor sequencing and disconnection	Global disconnection pattern with motor coordination impact

**Table 6:** Bootstrap-stable factor-derived MRI patterns using Oblimin rotation. The table shows the most stable representative variables for each factor, selected according to their frequency among the strongest absolute loadings across 1000 bootstrap iterations. Oblimin rotation allows factors to be correlated; therefore, these patterns should be interpreted as partially overlapping exploratory dimensions rather than independent biological networks. In the absence of external validation, these attributes should be considered exploratory. Abbreviations: FA, fractional anisotropy; MD, mean diffusivity; FLAIR, fluid-attenuated inversion recovery; Cereb, cerebellum; WM, white matter; Ctx, cortex; Thal, thalamus; LGN, lateral geniculate nucleus; CC, corpus callosum; ICV, intracranial volume; L/R, left/right; pons AP diam, anteroposterior pontine diameter; deg index, degeneration index; p90, 90th percentile; std, standard deviation; cv, coefficient of variation; normWM, normalized to white matter; thick avg, average cortical thickness; grayvol, gray matter volume; mm3, cubic millimeters.

Factor	Most Stable MRI-Derived Variables	Dominant Anatomical Domains	Descriptive MRI-Derived Pattern Interpretation
F1	FA_Cereb_WM_R_normWM; FA_Cereb_WM_R; FA_Cereb_WM_L; FA_Cereb_WM_L_normWM; FLAIR_occWM_z; MD_Brainstem	Cerebellar white matter, occipital white matter, brainstem, thalamus	Cerebellar white-matter integrity pattern with brainstem and thalamic diffusion involvement
F2	Motor_ICV; Cereb_ICV; Brainstem_ICV; Insula_ICV; Ventricle_4_mm3; Thal_ICV	Global cortical, cerebellar, brainstem, ventricular, and thalamic volumes	Global structural burden/intracranial-normalized volumetric pattern
F3	FA_Thal_L; Brainstem_deg_index; FLAIR_Cereb_WM_L_std; FLAIR_Thal_L; FLAIR_Thal_L_std; FLAIR_Thal_R_std	Thalamus, brainstem, cerebellar white matter, LGN	Thalamic–brainstem–cerebellar signal and microstructural pattern
F4	FA_Brainstem; Ventricle_3_mm3; Insula_L_thick_avg; Motor_R_thick_avg; CC_Posterior_mm3; Insula_R_thick_avg	Brainstem, ventricular system, insular/motor cortex, corpus callosum	Brainstem–cortical–callosal structural pattern
F5	FA_Brainstem_std; FA_LGN_R_std; pons_AP_diam_mm; FA_LGN_R; MD_LGN_R_std; MD_LGN_R	Brainstem, pons, lateral geniculate nucleus, white matter	Brainstem–pontine–visual relay microstructural pattern
F6	FLAIR_LGN_R_cv; MD_Thal_R_std; FLAIR_Thal_R_cv; MD_Thal_L_std; MD_Thal_R_normWM; FLAIR_Thal_R_std	LGN, bilateral thalamus, occipital white matter	Thalamic–visual relay diffusion/FLAIR heterogeneity pattern
F7	Insula_L_thick_avg; Insula_R_thick_avg; FA_Cereb_Ctx_L_normWM; FA_Cereb_Ctx_L; FLAIR_Thal_L_cv; FA_Brainstem_std	Insula, cerebellar cortex, thalamus, brainstem, LGN	Insular–cerebellar–thalamic sensory–motor pattern
F8	MD_Cereb_WM_L_std; MD_Cereb_WM_R_std; MD_Cereb_Ctx_L_std; FLAIR_Brainstem_std; FLAIR_Cereb_Ctx_L_std; FA_Cereb_Ctx_L_std	Cerebellar white matter/cortex, brainstem, thalamus	Cerebellar diffusion heterogeneity pattern with brainstem involvement
F9	FLAIR_Cereb_WM_L_cv; FLAIR_Cereb_WM_R_cv; FLAIR_Cereb_Ctx_R_cv; FLAIR_Cereb_Ctx_L_cv; FLAIR_Cereb_Ctx_L_std; FLAIR_LGN_R_cv	Cerebellar white matter/cortex, LGN, thalamus	Cerebellar FLAIR signal-heterogeneity pattern with visual relay involvement
F10	FLAIR_LGN_R_cv; FLAIR_LGN_R_std; FLAIR_occWM_z; FLAIR_Cereb_Ctx_L_cv; FLAIR_Thal_L_cv; FLAIR_Cereb_Ctx_R_std	LGN, occipital white matter, cerebellar cortex, thalamus, brainstem	Visual relay–occipital–cerebellar FLAIR heterogeneity pattern
F11	FLAIR_Thal_R_cv; FLAIR_Brainstem_cv; FLAIR_Thal_L_cv; Insula_R_thick_avg; FLAIR_Brainstem_std; FLAIR_Thal_L	Bilateral thalamus, brainstem, insula, LGN	Thalamic–brainstem FLAIR heterogeneity pattern with insular involvement
F12	FLAIR_Cereb_Ctx_R_std; FLAIR_Cereb_Ctx_L_std; FLAIR_Thal_L_std; FLAIR_occWM_z; FLAIR_Cereb_Ctx_R_cv; FLAIR_Brainstem_std	Cerebellar cortex, thalamus, occipital white matter, brainstem	Cerebellar–thalamic–occipital FLAIR signal-heterogeneity pattern

**Table 7:** MRI-derived features showing recurrent exploratory associations with neurological symptoms in Wolfram syndrome. For each MRI feature, the table reports the number of neurological manifestations associated with the feature, the mean absolute correlation across associated symptoms, and the maximum absolute correlation observed for an individual symptom. Higher values indicate MRI features with larger exploratory correlation values or more recurrent descriptive associations. Features associated with multiple symptoms may represent broader exploratory imaging correlates of neurological burden, whereas features with high maximum correlations may reflect possible symptom-specific imaging patterns. In the absence of external validation, these attributes should be interpreted as exploratory. Abbreviations: FA, fractional anisotropy; MD, mean diffusivity; FLAIR, fluid-attenuated inversion recovery; LGN, lateral geniculate nucleus; Thal, thalamus; Cereb, cerebellum; Ctx, cortex; WM, white matter; Brainstem, brainstem; CC, corpus callosum; Occip, occipital cortex; L/R, left/right; cv, coefficient of variation; p90, 90th percentile; std, standard deviation; mean, mean signal intensity; thick avg, average cortical thickness; normWM, normalized to white matter; mm3, cubic millimeters.

MRI Attribute	Number of Associated Neurological Manifestations	Mean Absolute Correlation	Maximum Absolute Correlation
FLAIR_LGN_L_cv	8	0.53	0.85
FLAIR_Thal_R_cv	8	0.53	0.85
FA_Cereb_Ctx_R	5	0.52	0.72
MD_LGN_R	8	0.47	0.72
FLAIR_Thal_L_cv	8	0.55	0.72
FA_Brainstem_std	5	0.52	0.72
FLAIR_LGN_R_p90	5	0.49	0.69
CC_Mid_Posterior_mm3	6	0.48	0.69
FLAIR_Cereb_Ctx_R_std	5	0.51	0.66
MD_LGN_L	8	0.45	0.66
Occip_R_thick_avg	10	0.50	0.66
FA_Cereb_Ctx_L	5	0.50	0.66
Occip_L_thick_avg	7	0.49	0.66
MD_Thal_R	8	0.45	0.66
MD_Cereb_Ctx_R_normWM	8	0.46	0.66
FLAIR_Cereb_Ctx_R	2	0.52	0.66
FLAIR_Cereb_Ctx_R_mean	2	0.52	0.66
FLAIR_Cereb_Ctx_R_cv	5	0.44	0.66
FLAIR_LGN_R_mean	5	0.47	0.66
FLAIR_LGN_R	5	0.47	0.66
FA_Cereb_WM_L	7	0.51	0.63
MD_Cereb_WM_R	6	0.48	0.63
MD_LGN_R_normWM	5	0.47	0.63
MD_Cereb_WM_L_normWM	7	0.48	0.63

**Table 8:** Exploratory structure–symptom associations between MRI-derived features and neurological manifestations in Wolfram syndrome. The table summarizes selected individual correlations identified in the symptom-associated correlation analysis. Positive associations indicate that higher MRI-derived values were associated with greater clinical involvement, whereas negative associations indicate that lower structural or microstructural integrity, such as reduced cortical thickness or fractional anisotropy, was associated with greater clinical involvement. In the absence of external validation, these attributes should be interpreted as exploratory. Abbreviations: LGN, lateral geniculate nucleus; FLAIR, fluid-attenuated inversion recovery; WM, white matter; corpus callosum, interhemispheric white-matter commissure.

Clinical Symptom	Main Associated MRI-Derived Features	Correlation Direction/Strength
Ataxia	LGN/thalamus FLAIR metrics; thalamic measures; cerebellar and parietal regions	Positive associations, up to r = 0.85
Dysphagia	Parietal cortex; insula; motor and occipital cortical measures	Negative associations, approximately r = −0.72 to −0.79
Gait and tandem gait impairment	Brainstem; cerebellar cortex; white-matter integrity metrics	Brainstem positive association around r = 0.72; cerebellar and white-matter integrity negative associations around r = −0.69 to −0.72
Dysmetria	Cerebellar white matter and lateral geniculate nucleus metrics	Negative associations around r = −0.66 to −0.69
Hyperreflexia	Corpus callosum and parietal cortical measures	Negative associations around r = −0.69 to −0.72
Anosmia	Cerebellar cortical measures	Negative association around r = −0.66

**Table 9:** MRI-derived features showing exploratory group-separation values according to dysphagia status. For each MRI-derived feature, the table reports the mean and standard deviation in patients without dysphagia (Mean 0 and SD 0) and in patients with dysphagia (Mean 1 and SD 1), together with the square root of the Fisher ratio used as a descriptive group-separability metric. Values should be interpreted as exploratory and not as formal hypothesis tests or validated diagnostic thresholds. In the absence of external validation, these attributes should be interpreted as exploratory. Abbreviations: FLAIR, fluid-attenuated inversion recovery; FA, fractional anisotropy; MD, mean diffusivity; LGN, lateral geniculate nucleus; Thal, thalamus; Cereb, cerebellum; WM, white matter; Brainstem, brainstem; pons, pons; CC, corpus callosum; Parietal, parietal cortex; Insula, insular cortex; Motor, motor cortex; Occip, occipital cortex; Ventricle_3, third ventricle; L/R, left/right; cv, coefficient of variation; std, standard deviation; thick avg, average cortical thickness; grayvol, gray matter volume; ICV, intracranial volume; ratio, anatomical ratio; normWM, normalized to white matter; mm3, cubic millimeters.

MRI-Derived Feature	Mean 0	SD 0	Mean 1	SD 1	$\sqrt{Fisher}$ *Ratio*
FLAIR_Thal_L_cv	0.11	0.01	0.13	0.01	1.32
Ventricle_3_mm3	997.7	302.6	1366.4	281.1	0.89
FLAIR_LGN_L_cv	0.11	0.01	0.12	0.02	0.83
FLAIR_Thal_R_cv	0.11	0.01	0.12	0.02	0.83
FLAIR_Thal_L_std	25.88	5.87	30.83	2.25	0.79
LGN_Thal_ratio_R	0.01	0.00	0.01	0.00	0.77
LGN_Thal_ratio_L	0.01	0.00	0.01	0.00	0.73
MD_Cereb_WM_L_std	78.7	33.9	104.9	25.9	0.62
Parietal_R_thick_avg	2.53	0.09	2.27	0.14	1.53
Insula_R_thick_avg	3.21	0.12	2.95	0.13	1.48
Motor_L_thick_avg	2.56	0.07	2.31	0.18	1.29
Parietal_L_thick_avg	2.51	0.11	2.26	0.17	1.29
Motor_R_thick_avg	2.53	0.11	2.33	0.14	1.13
Occip_R_thick_avg	2.12	0.10	1.96	0.12	1.06
Occip_L_thick_avg	2.08	0.09	1.95	0.09	1.05
Thal_ICV	15,679.5	2847.8	12,484.0	2126.6	0.90
Insula_L_thick_avg	3.25	0.17	3.03	0.17	0.88
CC_Mid_Posterior_mm3	552.9	71.9	434.7	114.6	0.87
Motor_R_grayvol_mm3	22,957.4	1899.9	19,926.7	2912.0	0.87
Thal_R_mm3	6273.54	916.6	5151.9	1021.1	0.82
Thal_L_mm3	6471.51	966.6	5279.9	1157.5	0.79
Brainstem_mm3	15,702.37	2361.2	13,165.2	2727.6	0.70
Brainstem_ICV	19,313.12	3563.8	15,865.2	3405.9	0.70
pons_vol_mm3	10,248.63	1493.8	8483.22	2108.7	0.68
FLAIR_Brainstem_std	31.85	6.1	36.8	4.71	0.64
FA_Cereb_WM_L	1481.43	244.6	1280.6	196.55	0.64
Parietal_L_grayvol_mm3	34,473.78	1867.1	29,689.3	7269.68	0.64

**Table 10:** MRI-derived imaging-feature categories associated with dysphagia status. The table summarizes the main MRI-derived alterations observed in patients with dysphagia, grouped by exploratory imaging-feature category. For each category, the principal MRI features, direction of change in the dysphagia group, descriptive biological interpretation, and relative exploratory relevance are reported. Lower cortical thickness, reduced subcortical and brainstem volumes, decreased cerebellar white-matter integrity, increased FLAIR heterogeneity, and ventricular enlargement are compatible with a distributed dysphagia-related MRI pattern involving cortical, thalamic, cerebellar, and brainstem systems. These categories should not be interpreted as validated biomarkers or disease-staging markers. In the absence of external validation, these attributes should be interpreted as exploratory. Abbreviations: L/R, left/right; GM, gray matter; FA, fractional anisotropy; MD, mean diffusivity; FLAIR, fluid-attenuated inversion recovery; WM, white matter; Cereb, cerebellum; LGN, lateral geniculate nucleus; CV, coefficient of variation; STD, standard deviation; Pons, pons; ICV, intracranial volume.

Exploratory Imaging-Feature Category	Main MRI-Derived Features	Direction in Dysphagia Group	Descriptive Interpretation	Relative Exploratory Relevance
Cortical thickness	Parietal L/R, Insula L/R, Motor L/R, Occipital L/R	Lower	Widespread cortical thinning compatible with greater cortical involvement	Very high
Subcortical and brainstem volume	Thalamus L/R, Brainstem, Pons	Lower	Reduced deep gray-matter and brainstem motor-bulbar hub volumes	High
Cortical gray-matter volume	Left parietal GM, Right motor GM	Lower	Localized cortical volume reduction	High
Interhemispheric connectivity	Mid-posterior corpus callosum volume	Lower	Reduced long-range cortical connectivity	High
Cerebellar white-matter integrity	FA_Cereb_WM_L	Lower	Possible cerebellar output tract involvement	Moderate-high
FLAIR heterogeneity in deep nuclei	Thalamus L/R CV and STD, LGN CV	Higher	Possible deep gray-matter signal heterogeneity	High, signal-based
Structural ratios	LGN/Thalamus ratios L/R	Higher	Possible disproportionate thalamic involvement	Moderate
Ventricular enlargement	Third ventricle volume	Higher	Possible secondary ventricular enlargement related to tissue loss	Moderate
Brainstem signal variability	FLAIR_Brainstem_std	Higher	Possible heterogeneous brainstem tissue involvement	Moderate
Cerebellar diffusion heterogeneity	MD_Cereb_WM_L_std	Higher	Possible non-uniform cerebellar white-matter microstructural change	Moderate

**Table 11:** MRI-derived features showing exploratory group-separation patterns according to ataxia status. For each MRI feature, the table reports the mean and standard deviation in patients with and without ataxia. The square root of the Fisher ratio was used as an exploratory measure of group separability, with higher values indicating greater descriptive separation between patients with and without ataxia. In the absence of external validation, these attributes should be interpreted as exploratory. Abbreviations: FLAIR, fluid-attenuated inversion recovery; Thal, thalamus; LGN, lateral geniculate nucleus; LatVent, lateral ventricle; Parietal, parietal cortex; Insula, insular cortex; Occip, occipital cortex; L/R, left/right; cv, coefficient of variation; ratio, anatomical ratio; thick avg, average cortical thickness; grayvol, gray matter volume; LR_asym, left–right asymmetry index; mm3, cubic millimeters.

MRI Feature	Mean 0	SD 0	Mean 1	SD 1	$\sqrt{Fisher}\;Ratio$
FLAIR_Thal_L_cv	0.11	0.01	0.13	0.01	1.32
FLAIR_LGN_L_cv	0.11	0.01	0.12	0.02	0.83
FLAIR_Thal_R_cv	0.11	0.01	0.12	0.02	0.83
LGN_Thal_ratio_R	0.01	0.00	0.01	0.00	0.77
LGN_Thal_ratio_L	0.01	0.00	0.01	0.00	0.73
LatVent_R_mm3	5759.9	2427.2	8859.8	5652.4	0.50
Parietal_R_thick_avg	2.53	0.09	2.27	0.14	1.53
Insula_R_thick_avg	3.21	0.12	2.95	0.13	1.48
Parietal_L_thick_avg	2.51	0.11	2.26	0.17	1.29
Occip_R_thick_avg	2.12	0.10	1.96	0.12	1.06
Occip_L_thick_avg	2.08	0.09	1.95	0.09	1.05
Insula_L_thick_avg	3.25	0.17	3.03	0.17	0.88
Thal_R_mm3	6273.5	916.6	5151.9	1021.1	0.82
Thal_L_mm3	6471.5	966.6	5279.9	1157.5	0.79
Parietal_LR_asym	−0.02	0.05	−0.09	0.08	0.79
Parietal_L_grayvol_mm3	34,473.8	1867.1	29,689.3	7269.7	0.64

**Table 12:** MRI-derived imaging-feature categories associated with ataxia status. This table groups ataxia-related MRI alterations by exploratory imaging-feature category, reporting the main features, direction of change, biological interpretation, and descriptive relevance. The pattern suggests possible combined cortical, thalamic, deep gray matter, and ventricular involvement. In the absence of external validation, these attributes should be interpreted as exploratory. Abbreviations: L/R, left/right; GM, gray matter; FLAIR, fluid-attenuated inversion recovery; Thal, thalamus; LGN, lateral geniculate nucleus; CV, coefficient of variation; LatVent, lateral ventricle.

Category	Main MRI-Derived Features	Direction in Ataxia Group	Descriptive Interpretation	Exploratory Relative Importance
Cortical thickness	Parietal L/R, insula L/R, occipital L/R	↓ Lower	Lower cortical thickness in multiple regions suggesting cortical involvement	Very high
Subcortical gray-matter volume	Thalamus L/R	↓ Lower	Lower thalamic volume with anatomically plausible involvement of sensory–motor relay regions	High
Cortical gray-matter volume	Left parietal gray matter	↓ Lower	Lower local cortical gray-matter volume	Moderate-high
Hemispheric asymmetry	Parietal left-right asymmetry	↓ Lower	Altered parietal asymmetry interpreted descriptively as a possible network-organization feature	Moderate
FLAIR heterogeneity in deep nuclei	Thalamus L/R CV, LGN L CV	↑ Higher	Possible deep gray-matter signal heterogeneity	High, signal-based
Structural ratios	LGN/thalamus ratios L/R	↑ Higher	Possible disproportionate thalamic/LGN relationship	Moderate
Ventricular enlargement	Right lateral ventricle	↑ Higher	Possible secondary expansion related to tissue loss	Moderate

**Table 13:** MRI-derived features showing exploratory group-separation patterns according to gait instability status. For each MRI feature, the table reports the mean and standard deviation in patients with and without gait instability. The square root of the Fisher ratio was used as an exploratory measure of group separability, with higher values indicating greater descriptive separation between patients with and without gait instability. In the absence of external validation, these attributes should be interpreted as exploratory. Abbreviations: FA, fractional anisotropy; FLAIR, fluid-attenuated inversion recovery; Thal, thalamus; LGN, lateral geniculate nucleus; LatVent, lateral ventricle; Ventricle_3, third ventricle; Parietal, parietal cortex; Insula, insular cortex; Motor, motor cortex; Occip, occipital cortex; occWM, occipital white matter; Cereb, cerebellum; WM, white matter; L/R, left/right; cv, coefficient of variation; z, z-score; thick avg, average cortical thickness; mean, mean value; mm3, cubic millimeters.

MRI Feature	Mean 0	SD 0	Mean 1	SD 1	$\sqrt{Fisher}\;Ratio$
FLAIR_Thal_L_cv	0.11	0.01	0.13	0.01	1.32
Ventricle_3_mm3	997.7	302.6	1366.4	281.1	0.89
FLAIR_Thal_R_cv	0.11	0.01	0.12	0.02	0.83
FLAIR_LGN_L_cv	0.11	0.01	0.12	0.02	0.83
LatVent_R_mm3	5759.9	2427.2	8859.8	5652.4	0.50
Parietal_R_thick_avg	2.53	0.09	2.27	0.14	1.53
Insula_R_thick_avg	3.21	0.12	2.95	0.13	1.48
Motor_L_thick_avg	2.56	0.07	2.31	0.18	1.29
Parietal_L_thick_avg	2.51	0.11	2.26	0.17	1.29
Occip_R_thick_avg	2.12	0.10	1.96	0.12	1.06
Occip_L_thick_avg	2.08	0.09	1.95	0.09	1.05
Insula_L_thick_avg	3.25	0.17	3.03	0.17	0.88
FLAIR_occWM_z	0.34	0.12	0.17	0.19	0.75
FA_Cereb_WM_L	1481.4	244.6	1280.6	196.5	0.64
FA_WM_mean	1500.4	142.0	1410.5	79.8	0.55

**Table 14:** MRI-derived imaging-feature categories associated with gait instability status. The table groups gait-instability-related MRI alterations by exploratory imaging-feature category, reporting the main features, direction of change, biological interpretation, and descriptive relevance. The pattern suggests possible combined cortical, deep gray matter, white-matter, and ventricular involvement. In the absence of external validation, these attributes have an exploratory character. Abbreviations: L/R, left/right; FA, fractional anisotropy; FLAIR, fluid-attenuated inversion recovery; WM, white matter; Cereb, cerebellum; Thal, thalamus; LGN, lateral geniculate nucleus; CV, coefficient of variation; LatVent, lateral ventricle; Ventricle_3, third ventricle; occWM, occipital white matter; z, z-score.

Category	Main MRI-Derived Features	Direction in Gait-Instability Group	Descriptive Interpretation	Exploratory Relative Importance
Cortical thickness	Parietal L/R, insula L/R, motor L, occipital L/R	↓ Lower	Lower cortical thickness in multiple regions suggesting cortical involvement	Very high
White-matter integrity	FA_Cereb_WM_L, FA_WM_mean	↓ Lower	Lower cerebellar and global white-matter integrity measures	High
FLAIR heterogeneity in deep nuclei	Thalamus L/R CV, LGN L CV	↑ Higher	Possible deep gray-matter signal heterogeneity	High, signal-based
Ventricular enlargement	Third ventricle, right lateral ventricle	↑ Higher	Possible secondary expansion related to tissue loss	Moderate
FLAIR signal in posterior white matter	FLAIR_occWM_z	↓ Lower	Altered posterior white-matter signal relative to the reference measure	Moderate

**Table 15:** MRI-derived features showing exploratory group-separation values according to cognitive impairment status. For each MRI-derived feature, the table reports the mean and standard deviation in patients without cognitive impairment and patients with cognitive impairment. The square root of the Fisher ratio was used as a descriptive group-separability metric, with higher values indicating greater descriptive separation between groups. In the absence of external validation, these attributes should be interpreted as exploratory. Abbreviations: FA, fractional anisotropy; FLAIR, fluid-attenuated inversion recovery; Thal, thalamus; LGN, lateral geniculate nucleus; Cereb, cerebellum; WM, white matter; Brainstem, brainstem; pons, pons; CC, corpus callosum; Insula, insular cortex; Motor, motor cortex; Parietal, parietal cortex; LatVent, lateral ventricle; Ventricles_Total, total ventricular volume; L/R, left/right; cv, coefficient of variation; std, standard deviation; ratio, anatomical ratio; thick avg, average cortical thickness; grayvol, gray matter volume; LR_asym, left–right asymmetry index; occWM, occipital white matter; z, z-score; mm3, cubic millimeters.

MRI Feature	Mean 0	SD 0	Mean 1	SD 1	$\sqrt{Fisher}\;Ratio$
FLAIR_Thal_L_cv	0.11	0.01	0.13	0.01	1.32
FLAIR_Thal_R_cv	0.11	0.01	0.12	0.02	0.83
FLAIR_LGN_L_cv	0.11	0.01	0.12	0.02	0.83
LGN_Thal_ratio_R	0.01	0.00	0.01	0.00	0.77
FLAIR_LGN_L_std	24.9	4.8	29.4	4.3	0.70
FLAIR_Thal_R_std	24.9	4.8	29.4	4.3	0.70
LatVent_L_mm3	6531.9	2450.9	9028.2	3788.7	0.55
Ventricles_Total_mm3	15,393.3	5467.7	21,397.3	9691.9	0.54
LatVent_R_mm3	5759.9	2427.2	8859.8	5652.4	0.50
Insula_R_thick_avg	3.21	0.12	2.95	0.13	1.48
Motor_L_thick_avg	2.56	0.07	2.31	0.18	1.29
CC_Mid_Posterior_mm3	552.9	72.0	434.7	114.6	0.87
Motor_R_grayvol_mm3	22,957.4	1900.0	19,926.7	2912.0	0.87
Thal_R_mm3	6273.5	916.6	5151.9	1021.1	0.82
Parietal_LR_asym	−0.02	0.05	−0.09	0.08	0.79
FLAIR_occWM_z	0.34	0.12	0.17	0.19	0.75
Brainstem_mm3	15,702.4	2361.2	13,165.2	2727.6	0.70
pons_vol_mm3	10,248.6	1493.8	8483.2	2108.7	0.68
FA_Cereb_WM_L	1481.4	244.6	1280.6	196.5	0.64
Parietal_L_grayvol_mm3	34,473.8	1867.1	29,689.3	7269.7	0.64
FA_Cereb_WM_R	1518.6	230.3	1324.2	256.8	0.56
Cereb_WM_L_mm3	11,207.7	1223.1	9755.2	2557.4	0.51

**Table 16:** Summary of exploratory MRI-derived feature categories associated with cognitive impairment status. The table groups cognitive-impairment-related MRI alterations by exploratory imaging-feature category, reporting the main features, direction of change, biological interpretation, and relative importance. In the absence of external validation, these attributes should be interpreted as exploratory. Abbreviations: L/R, left/right; GM, gray matter; FA, fractional anisotropy; FLAIR, fluid-attenuated inversion recovery; WM, white matter; Cereb, cerebellum; Thal, thalamus; LGN, lateral geniculate nucleus; CV, coefficient of variation; STD, standard deviation; CC, corpus callosum; LR asymmetry, left–right asymmetry index; occWM, occipital white matter; z, z-score.

Category	Main MRI-Derived Features	Direction in Cognitive-Impairment Group	Interpretation	Exploratory Relative Importance
Cortical thickness and gray-matter volume	Insula R, Motor L, Parietal GM L	↓ Lower	Cortical thinning and lower gray-matter volume suggesting cortical involvement	Very High
Subcortical and brainstem volume	Thalamus R, Brainstem, Pons	↓ Lower	Lower deep gray-matter and brainstem volumes involving motor-bulbar hubs	High
Interhemispheric connectivity	Mid-posterior corpus callosum volume	↓ Lower	Reduced long-range cortical connectivity	High
Cerebellar white-matter integrity	FA_Cereb_WM L/R, Cerebellar WM volume L	↓ Lower	Possible cerebellar output pathway involvement	Moderate-High
Hemispheric asymmetry	Parietal LR asymmetry	↓ Lower	Altered hemispheric balance and network organization	Moderate
FLAIR heterogeneity in deep nuclei	Thalamus L/R CV and STD, LGN CV and STD	↑ Higher	Possible deep gray matter tissue heterogeneity	High, signal-based
Ventricular enlargement	Lateral ventricles L/R, total ventricles	↑ Higher	Possible secondary expansion related to tissue loss	Moderate
Posterior white-matter FLAIR signal	FLAIR_occWM_z	↓ Lower	Altered posterior white-matter signal	Moderate

**Table 17:** Exploratory MRI-derived feature categories associated with neurological burden. The table summarizes MRI-derived feature categories according to their cross-sectional association with neurological burden in Wolfram syndrome. These categories are descriptive and should not be interpreted as temporal disease stages, progression markers, or validated biomarkers. In the absence of external validation in larger populations, these findings should be considered exploratory. Abbreviations: FA, fractional anisotropy; FLAIR, fluid-attenuated inversion recovery; LGN, lateral geniculate nucleus; CV, coefficient of variation; STD, standard deviation; WM, white matter; GM, gray matter; L/R, left/right; CC, corpus callosum.

MRI-Derived Feature Pattern	MRI Feature Category	Key MRI-Derived Attributes	Descriptive Interpretation
Signal-based deep gray-matter pattern	Deep gray-matter FLAIR heterogeneity	Thalamic and LGN FLAIR CV/STD	Tissue heterogeneity, gliosis, demyelination, or microstructural stress
Ventricular pattern	Ventricular enlargement	Third ventricle, lateral ventricles	Possible indirect correlate of central tissue loss or global structural burden
Cortical integration pattern	Cortical thinning in association cortex	Parietal, insular, and occipital thickness	Cortical involvement affecting integration networks
White-matter integrity pattern	White-matter disruption	Cerebellar and global FA reduction	Disruption of long-range motor and cerebellar pathways
Thalamic relay pattern	Thalamic volume loss	Thalamus L/R	Involvement of sensory–motor relay hubs
Motor cortical pattern	Motor cortical structural alteration	Motor cortex thickness and gray-matter volume	Involvement of motor execution networks
Brainstem–pontine pattern	Brainstem and pons volume reduction	Brainstem volume, pons volume	Involvement of bulbar, autonomic, and motor pathways
Callosal pattern	Interhemispheric white-matter involvement	Mid-posterior corpus callosum volume	Altered interhemispheric and long-range connectivity
Cerebellar output pattern	Cerebellar white-matter involvement	Cerebellar WM FA L/R, cerebellar WM volume	Involvement of cerebellar output pathways

**Table 18:** Structural and signal-based MRI-derived feature domains associated with neurological burden. The table summarizes two broad MRI-derived feature domains associated with neurological burden. Structural alterations and signal-based tissue alterations are presented as complementary exploratory patterns. They should not be interpreted as validated biomarkers, temporal progression markers, or disease-staging categories. In the absence of external validation in larger populations, these findings should be considered exploratory. Abbreviations: FA, fractional anisotropy; FLAIR, fluid-attenuated inversion recovery; LGN, lateral geniculate nucleus; WM, white matter; GM, gray matter.

MRI-Derived Feature Domain	Main MRI-Derived Alterations	Exploratory Role in Neurological Burden	Descriptive Interpretation
Structural alterations	Cortical thinning in parietal, insular, motor, and occipital regions	Structural component of neurological burden	Involvement of cortical motor and integration networks
Structural alterations	Motor and parietal gray-matter volume loss	Structural component of neurological burden	Cortical tissue loss associated with functional impairment
Structural alterations	Lower thalamic volume	Subcortical structural component	Involvement of sensory–motor relay nuclei
Structural alterations	Brainstem and pons volume reduction	Brainstem–bulbar structural component	Involvement of bulbar, autonomic, and motor pathways
Structural alterations	Mid-posterior corpus callosum reduction	Connectivity-related structural component	Interhemispheric and long-range connectivity involvement
Structural alterations	Cerebellar white-matter FA and volume loss	Cerebellar structural component	Involvement of cerebellar output pathways
Signal-based tissue alterations	Increased FLAIR heterogeneity in thalamus and LGN	Complementary tissue-contrast pattern	Deep gray-matter tissue heterogeneity, gliosis, or demyelination
Signal-based tissue alterations	Increased diffusion variability in deep nuclei and cerebellar white matter	Complementary microstructural pattern	Microstructural heterogeneity and pathway disruption
Signal-based tissue alterations	Increased third, lateral, and total ventricular volumes	Complementary structural-burden pattern	Secondary enlargement related to tissue loss; temporal meaning cannot be inferred
Signal-based tissue alterations	Altered posterior white-matter FLAIR signal	Complementary posterior white-matter pattern	Posterior white-matter signal abnormality related to network involvement

## Data Availability

The data presented in this study are available from the corresponding author upon reasonable request, subject to ethical, legal, and privacy restrictions.

## Supplementary Materials

The following supporting information can be downloaded at: https://www.mdpi.com/article/10.3390/diagnostics16152396/s1, Table S1: PCA-derived MRI pattern interpretation; Table S2: Factor-derived MRI network interpretation.

## References

[1] Barrett T.G., Bundey S.E., Macleod A.F. (1995). Neurodegeneration and diabetes: UK nationwide study of Wolfram (DIDMOAD) syndrome. Lancet.

[2] Rigoli L., Di Bella C. (2012). Wolfram syndrome 1 and Wolfram syndrome 2. Curr. Opin. Pediatr..

[3] Urano F. (2016). Wolfram Syndrome: Diagnosis, Management, and Treatment. Curr. Diab. Rep..

[4] Esteban-Bueno G., Ruiz-Castañeda D., Ruiz Martínez J., Romero Muñoz M., Carrillo Alascio P. (2018). Natural history and clinical characteristics of 50 patients with Wolfram syndrome. Endocrine.

[5] Chaussenot A., Bannwarth S., Rouzier C., Vialettes B., Mkadem S.A., Chabrol B., Cano A., Labauge P., Paquis-Flucklinger V. (2011). Neurologic features and genotype-phenotype correlation in Wolfram syndrome. Ann. Neurol..

[6] Marshall B.A., Permutt M.A., Paciorkowski A.R., Hoekel J., Karzon R., Wasson J., Viehover A., White N.H., Shimony J.S., Manwaring L. (2013). Phenotypic characteristics of early Wolfram syndrome. Orphanet J. Rare Dis..

[7] Rohayem J., Ehlers C., Wiedemann B., Holl R., Oexle K., Kordonouri O., Salzano G., Meissner T., Burger W., Schober E. (2011). Diabetes and neurodegeneration in Wolfram syndrome: A multicenter study of phenotype and genotype. Diabetes Care.

[8] de Heredia M.L., Clèries R., Nunes V. (2013). Genotypic classification of patients with Wolfram syndrome: Insights into the natural history of the disease and correlation with phenotype. Genet. Med..

[9] Inoue H., Tanizawa Y., Wasson J., Behn P., Kalidas K., Bernal-Mizrachi E., Mueckler M., Marshall H., Donis-Keller H., Crock P. (1998). A gene encoding a transmembrane protein is mutated in patients with diabetes mellitus and optic atrophy (Wolfram syndrome). Nat. Genet..

[10] Strom T.M., Hörtnagel K., Hofmann S., Gekeler F., Scharfe C., Rabl W., Gerbitz K.D., Meitinger T. (1998). Diabetes insipidus, diabetes mellitus, optic atrophy and deafness (DIDMOAD) caused by mutations in a novel gene (wolframin) coding for a predicted transmembrane protein. Hum. Mol. Genet..

[11] Amr S., Heisey C., Zhang M., Xia X.J., Shows K.H., Ajlouni K., Pandya A., Satin L.S., El-Shanti H., Shiang R. (2007). A homozygous mutation in a novel zinc-finger protein, ERIS, is responsible for Wolfram syndrome 2. Am. J. Hum. Genet..

[12] Kõks S. (2023). Genomics of Wolfram Syndrome 1 (WFS1). Biomolecules.

[13] Frontino G., Delvecchio M., Prudente S., Sordi V.D., Barboni P., Di Giamberardino A., Rutigliano A., Pellegrini S., Caretto A., Cascavilla M.L. (2025). SID/SIEDP expert consensus on optimizing clinical strategies for early detection and management of Wolfram syndrome. J. Endocrinol. Investig..

[14] Hershey T., Lugar H.M., Shimony J.S., Rutlin J., Koller J.M., Perantie D.C., Paciorkowski A.R., Eisenstein S.A., Permutt M.A., the Washington University Wolfram Study Group Early Brain Vulnerability in Wolfram Syndrome (2012). Early brain vulnerability in Wolfram syndrome. PLoS ONE.

[15] Lugar H.M., Koller J.M., Rutlin J., Marshall B.A., Kanekura K., Urano F., Bischoff A.N., Shimony J.S., Hershey T., the Washington University Wolfram Syndrome Research Study Group (2016). Neuroimaging evidence of deficient axon myelination in Wolfram syndrome. Sci. Rep..

[16] Hoekel J., Narayanan A., Rutlin J., Lugar H., Al-Lozi A., Hershey T., Tychsen L. (2018). Visual pathway function and structure in Wolfram syndrome: Patient age, variation and progression. BMJ Open Ophthalmol..

[17] Lugar H.M., Koller J.M., Rutlin J., Eisenstein S.A., Neyman O., Narayanan A., Chen L., Shimony J.S., Hershey T. (2019). Evidence for altered neurodevelopment and neurodegeneration in Wolfram syndrome using longitudinal morphometry. Sci. Rep..

[18] Samara A., Rahn R., Neyman O., Park K.Y., Samara A., Marshall B., Dougherty J., Hershey T. (2019). Developmental hypomyelination in Wolfram syndrome: New insights from neuroimaging and gene expression analyses. Orphanet J. Rare Dis..

[19] Samara A., Lugar H.M., Hershey T., Shimony J.S. (2020). Longitudinal assessment of neuroradiologic features in Wolfram syndrome. Am. J. Neuroradiol..

[20] Simo J., Lugar H.M., Miller E., Wilf-Yarkoni A., Goldberg Y., Kocaağa A., Ito S., Cocozza S., Frontino G., Baldoli C. (2025). Expanding the spectrum of white matter abnormalities in Wolfram syndrome: A retrospective review. Front. Neurol..

[21] Basser P.J., Mattiello J., LeBihan D. (1994). MR diffusion tensor spectroscopy and imaging. Biophys. J..

[22] Gillies R.J., Kinahan P.E., Hricak H. (2016). Radiomics: Images are more than pictures, they are data. Radiology.

[23] Zwanenburg A., Vallières M., Abdalah M.A., Aerts H.J.W.L., Andrearczyk V., Apte A., Ashrafinia S., Bakas S., Beukinga R.J., Boellaard R. (2020). The Image Biomarker Standardization Initiative: Standardized quantitative radiomics for high-throughput image-based phenotyping. Radiology.

[24] Shi M.G., Feng X.M., Zhi H.Y., Hou L., Feng D.F. (2024). Machine learning-based radiomics in neurodegenerative and cerebrovascular disease. MedComm.

[25] Lin G., Chen W., Geng Y., Peng B., Liu S., Chen M., Pang C., Chen P., Lu C., Yan Z. (2025). A multimodal MRI-based machine learning framework for classifying cognitive impairment in cerebral small vessel disease. Sci. Rep..

