# Supplementary material for: Exploratory Associations Between Multimodal MRI-Derived Features and Neurological Symptoms in Wolfram Syndrome: A Spanish Cohort Pilot Study
Source: Diagnostics (Basel). 2026 Jul 30;16(15):2396. doi: 10.3390/diagnostics16152396 (PMC13465571; doi:10.3390/diagnostics16152396)
Supplement: Supplementary file 1 [file diagnostics-16-02396-s001.zip › diagnostics-4395482-supplementary.pdf]

Supplementary Material

Supplementary Table S1. PCA-derived MRI pattern interpretation. Principal component labels were assigned by examining the MRI-derived attributes with the highest absolute loadings within each principal component. Because PCA represents a linear transformation of the MRI-derived attribute space, each principal component was interpreted as a weighted combination of the original attributes rather than as a predefined biological network. The contributing attributes were grouped according to their anatomical, tissue-level, and imaging-feature convergence, allowing each principal component to be described as an exploratory MRI-derived pattern. These interpretations should not be considered validated neurobiological networks and may change as additional harmonized MRI datasets become available.

PC	Main contributing variables	Network interpretation
PC1	Occipital gray volume, cerebellar WM volume, thalamus, peduncle, parietal/motor cortex, pons, corpus callosum	Global posterior-cerebellar-thalamic structural network
PC2	Occipital WM MD, thalamic FA/FLAIR, cerebellar WM FA, cerebellar FLAIR, insula thickness, brainstem FA	Thalamic-cerebellar-occipital white-matter diffusion network
PC3	Cerebellum/ICV, insula/ICV, 4th ventricle, thalamic MD/FA, thalamus/ICV	Global volume / ventricular-thalamic-cerebellar network
PC4	Occipital WM FLAIR, motor asymmetry, occipital asymmetry, LGN FLAIR, thalamic FLAIR, brainstem FA	Visual-motor asymmetry and FLAIR network
PC5	Cerebellar cortex MD, brainstem MD, LGN FA, occipital WM FA, cerebellar cortex FA/FLAIR	Cerebellar-brainstem-LGN diffusion network
PC6	Anterior corpus callosum, motor asymmetry, cerebellar cortex FA, brainstem FLAIR, cerebellar WM FLAIR, peduncle	Motor-cerebellar-brainstem/callosal network
PC7	LGN MD/FA, posterior corpus callosum, thalamic asymmetry, 3rd ventricle, brainstem FLAIR, pons	LGN-thalamic-callosal/ventricular network
PC8	Brainstem degeneration index, cerebellar cortex/WM ratio, parietal asymmetry, brainstem FLAIR, thalamus/ICV	Brainstem-cerebellar-parietal sensory-integration network
PC9	3rd ventricle, posterior/mid-anterior corpus callosum, LGN FLAIR, thalamic FA, occipital/insula volumes	Callosal-ventricular-visual/thalamic network
PC10	Cerebellar cortex FA, occipital asymmetry, parietal volume/asymmetry, pons FA, occipital WM FLAIR, brainstem MD	Cerebellar-parietal-occipital/brainstem network
PC11	Insula gray volume, pons FA, parietal asymmetry, cerebellar cortex/WM ratio, thalamus/ICV	Insula-pons-parietal sensorimotor network
PC12	Thalamic FA, insula thickness, anterior corpus callosum, cerebellar WM FA/FLAIR, 4th ventricle	Thalamo-insular-callosal/cerebellar WM network

PC	Main contributing variables	Network interpretation
PC13	Thalamic FA, corpus callosum, cerebellar WM FLAIR, pons diameter, LGN MD/FLAIR, motor cortex, peduncle	Thalamo-callosal-brainstem/LGN-motor network
PC14	Occipital WM FA, occipital gray volume, thalamic asymmetry, cerebellar WM FA/MD, thalamic FLAIR, pons	Occipital-visual/thalamic asymmetry network

Supplementary Table S2. Factor-derived MRI network interpretation. Factor-derived labels were assigned by examining the MRI-derived attributes with the highest absolute factor loadings within each factor. Because factor analysis represents a linear transformation of the MRI-derived attribute space, each factor was interpreted as a weighted combination of the original attributes rather than as a predefined latent MRI network. The contributing attributes were grouped according to their anatomical, tissue-level, and imaging-feature convergence, allowing each factor to be described as an exploratory MRI-derived pattern. These interpretations should not be considered validated neurobiological networks or stable latent constructs and may change as additional harmonized MRI datasets become available.

**Factor**	**Main contributing variables**	**Network interpretation**
F1	LGN FA/MD measures, pons diameter, brainstem FA, peduncle volume	LGN / visual-brainstem white-matter integrity factor
F2	Cerebellar WM FA, thalamic FA/MD, cerebellar cortex MD, occipital WM FA	Thalamic-cerebellar-white matter diffusion factor
F3	Brainstem MD, brainstem MD standard deviation, brainstem FLAIR CV, thalamic FLAIR, occipital WM MD	Brainstem diffusivity / brainstem injury factor
F4	Thalamus volume, thalamus/ICV, motor cortex, corpus callosum, cerebellum	Thalamo-motor/cerebellar structural factor
F5	Anterior corpus callosum, ventricles, lateral ventricle, insula/ICV	Ventricular / callosal / global atrophy factor
F6	Brainstem degeneration index, cerebellar cortex-WM ratio, thalamic FA, parietal asymmetry	Brainstem-cerebellar degeneration factor
F7	LGN FLAIR, motor asymmetry, occipital/parietal measures	Visual-motor asymmetry factor
F8	Cerebellar WM FLAIR CV, cerebellar cortex FLAIR CV, thalamic FLAIR	Cerebellar FLAIR / cerebellar signal-abnormality factor
F9	Posterior/mid-anterior corpus callosum, third ventricle, brainstem FA	Callosal-ventricular structural factor
F10	Thalamus asymmetry, brainstem FLAIR, occipital WM FA/MD, peduncle	Thalamic asymmetry + brainstem/occipital white-matter factor
F11	Insula gray volume, pons FA, occipital WM FLAIR, motor cortex	Insula-pons-motor factor
F12	Cerebellar cortex FA, occipital asymmetry, insula/parietal thickness/asymmetry	Cerebellar-cortical sensory-integration factor
